# Influence of Excess Charge on Water Adsorption on the BiVO_4_(010) Surface

**DOI:** 10.1021/jacs.2c07501

**Published:** 2022-09-08

**Authors:** Wennie Wang, Marco Favaro, Emily Chen, Lena Trotochaud, Hendrik Bluhm, Kyoung-Shin Choi, Roel van de Krol, David E. Starr, Giulia Galli

**Affiliations:** † Pritzker School of Molecular Engineering, University of Chicago, Chicago, Illinois 60637, United States; ‡ Institute for Solar Fuels, Helmholtz-Zentrum Berlin für Materialien und Energie GmbH, Hahn-Meitner-Platz 1, Berlin 14109, Germany; § Department of Chemistry, University of Chicago, Chicago, Illinois 60615, United States; ∥ Chemical Sciences Division, Lawrence Berkeley National Laboratory, Berkeley, California 94720, United States; ⊥ Department of Chemistry, University of Wisconsin−Madison, Madison, Wisconsin 53706, United States; # Institut für Chemie, Technische Universität Berlin, Straße des 17. Juni 124, Berlin 10623, Germany; ¶ Materials Science Division and Center for Molecular Engineering, Argonne National Laboratory, Lemont, Illinois 60439, United States

## Abstract

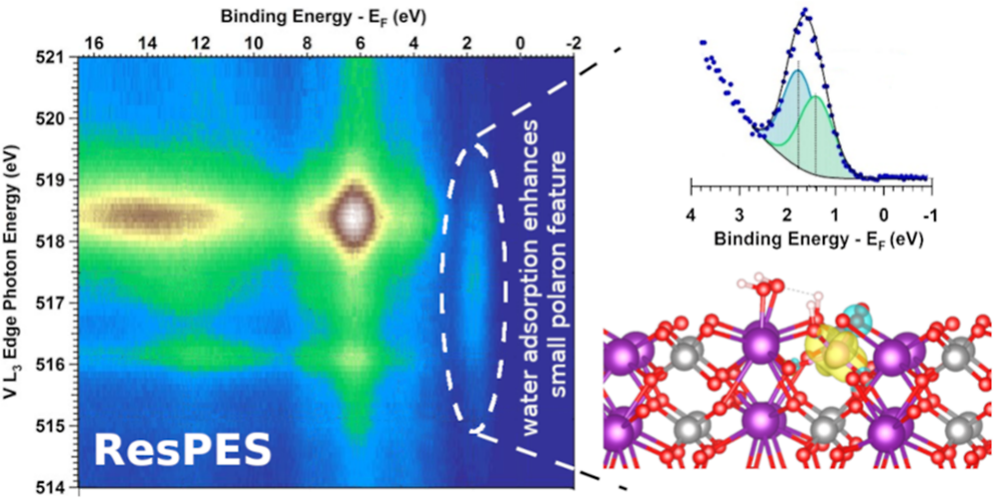

We present a combined computational and experimental study of the adsorption of water on the Mo-doped BiVO_4_(010) surface, revealing how excess electrons influence the dissociation of water and lead to hydroxyl-induced alterations of the surface electronic structure. By comparing ambient pressure resonant photoemission spectroscopy (AP-ResPES) measurements with the results of first-principles calculations, we show that the dissociation of water on the stoichiometric Mo-doped BiVO_4_(010) surface stabilizes the formation of a small electron polaron on the VO_4_ tetrahedral site and leads to an enhanced concentration of localized electronic charge at the surface. Our calculations demonstrate that the dissociated water accounts for the enhanced V^4+^ signal observed in ambient pressure X-ray photoelectron spectroscopy and the enhanced signal of a small electron polaron inter-band state observed in AP-ResPES measurements. For ternary oxide surfaces, which may contain oxygen vacancies in addition to other electron-donating dopants, our study reveals the importance of defects in altering the surface reactivity toward water and the concomitant water-induced modifications to the electronic structure.

## 1 Introduction

Using solar energy to split water and produce hydrogen fuel is an attractive avenue toward realizing a clean energy future,^1−5^ particularly in mitigating solar radiance fluctuations by storing solar energy in chemical bonds. Bismuth vanadate (BiVO_4_) is at the vanguard of complex oxide photoanode materials due to several advantageous properties, including high electron–hole separation efficiencies (over 70%) and photocurrent onset potentials very close to the thermodynamic hydrogen evolution potential.^6^ While the band gap of BiVO_4_ (∼2.4–2.6 eV^6−9^) is larger than desired, coupling it with smaller band gap materials^10^ and doping^6^ are promising strategies for enhancing light absorption. Doping with W or Mo also improves the intrinsically limiting carrier transport and separation efficiencies in BiVO_4_.^11−13^ These aspects combined with the ease and low cost of BiVO_4_ synthesis^14^ and its corrosion resistance^15^ have led to intense investigations and optimization of BiVO_4_ photoanodes for water splitting.^10,14,16−18^ However, despite the intensity and vast number of studies in the literature, a fundamental understanding of how the surface of BiVO_4_ interacts with water remains elusive.

At the semiconducting oxide/aqueous electrolyte interface, water may adsorb non-dissociatively (i.e., molecular water adsorption) or dissociatively, resulting in the hydroxylation of the surface. Determining when and to what extent dissociative water adsorption occurs along with the structural moieties involved is critical for understanding the changes in the electronic structure upon interface formation and therefore charge transfer across the interface. Water adsorption on binary semiconducting oxides has been studied extensively,^19−22^ and mechanistic insights into the interaction between the semiconductor surface and water have been revealed. For example, studies have shown that surface defects play a key role in water dissociation on TiO_2_(110) surfaces,^23^ though whether and to what extent water dissociates on the pristine rutile TiO_2_(110) surface have been controversial for both the experiment and theory.^23−28^ Nevertheless, these studies provide insights into the geometric configurations required for accurate electronic structural models and the intermediate species needed to simulate the mechanisms by which water splitting occurs. The influence of polarons on the structural, chemical, and electronic properties of the TiO_2_(110) surface as well as a comparison of their localization in rutile and anatase TiO_2_ has been extensively studied.^29−33^ These studies indicate that excess electrons and defects play a key role in the adsorption state of adsorbates on TiO_2_ surfaces and that adsorbates may modify the surface electronic structure by localizing excess electrons into polaron states.

Compared to binary oxides, however, very little is known about the exact nature of the interface between BiVO_4_ and water; for example, whether water dissociates on the defect-free surface or if a defective surface is required, which structural moieties are involved if dissociation does occur, and how the electronic structure of the BiVO_4_ surface is modified by water dissociation and hydroxylation. Thus far, a limited number of studies have examined the electronic properties of the (pristine) BiVO_4_ surface or its modification following (i.e., ex situ) exposure to water, with several of them using polycrystalline samples^34−36^ and a few of them using a combined experimental and computational framework.^37^ Of note are the soft X-ray spectroscopic studies by Jovic et al.^38,39^ who observed charge localization of excess electrons in inter-band gap small polaron states for W- and Mo-doped BiVO_4_ crystals, and the thorough studies by Favaro et al.^8^ on the chemical, structural, and electronic characteristics of the Mo-doped BiVO_4_(010) surface. While there is a general agreement that the BiVO_4_ surface is reduced with exposure to water,^35,36^ the exact structure and composition of the surface and the role of defects remain unclear.^34−36^ A number of computational studies have investigated the interaction of water with different surfaces of BiVO_4_. Interestingly, thus far, none have reported dissociation of water molecules, including the first-principles molecular dynamics (MD) simulations of Oshikiri and Boero, who simulated the adsorption of up to a monolayer (ML) of molecular water on the (100) surface of undoped BiVO_4_.^40^ The lowest energy (010) surface (in the *C*2/*c* convention)^41^ has been shown to have a similar behavior. For example, Yang and colleagues used density functional theory (DFT) calculations at the level of generalized gradient approximation and found that the adsorption of dissociated water is endothermic on the (undoped) (010) surface.^42^ Crespo-Otero and Walsh also studied the undoped (010) surface and used MD simulations based on the PBE and PBEsol functionals to study variations in the surface ionization potential [i.e., valence band (VB) edge] of the hydrated surface with temperature.^43^ Still, no dissociation of water on the surface was observed in their models for either a monolayer (ML) of water or liquid water. Recently, Wiktor and Pasquarello used first-principles MD to study charge-doped surfaces interfaced with molecular water.^44^ Interestingly, they reported that the electron polaron was less stable at the interface with liquid water compared to the non-hydrated surface, while the hole polaron was more stable; these results suggest that hydration can enhance electron–hole separation. This study likewise did not report the occurrence or adsorption of dissociated water. The discrepancy between the results found in the literature naturally raises the question whether dissociative water adsorption is possible on the defect-free, undoped surface of BiVO_4_ and what role surface defects, in particular excess electrons, play in water adsorption and dissociation on the BiVO_4_ surface. Understanding this fundamental aspect of the semiconductor/aqueous electrolyte interface has important implications for formulating a detailed mechanism for the oxygen evolution reaction in water splitting and a molecular-level model of its initial steps.^45^

To the best of our knowledge, we present for the first time a combined experimental and computational study that identifies species important for the hydroxylation of the BiVO_4_(010) surface and consequently the nature of water adsorption on the BiVO_4_ surface. Notably, we present a near one-to-one comparison of the electronic structure of single-crystalline samples and first-principles calculations. Ambient pressure X-ray photoelectron spectroscopy (AP-XPS) has been used extensively in the past to study the adsorption and dissociation of water on oxide surfaces.^46−51^ AP-XPS provides the ability to study water adsorption at room temperature (r.t.) in elevated water vapor pressures, conditions that closely simulate those found in the environment. These studies have provided detailed models of the hydroxylation of oxide surfaces as a function of relative humidity. Here, we have extended these types of studies to include resonant excitation. The utility of resonant photoemission spectroscopy (ResPES) as a means to identify elemental and orbital contributions of the occupied electronic states is highlighted, including in cases when weaker contributions are ordinarily difficult to disentangle from the stronger ones (as in the case of V 3d orbitals in the VBs of vanadium oxides that are dominated by the O 2p states). In particular, we demonstrate how ambient pressure resonant photoemission spectroscopy (AP-ResPES) may be used in conjunction with first-principles calculations to understand polaron formation in the presence of adsorbed and dissociated water on the BiVO_4_(010) surface. Our ResPES measurements reveal an enhanced peak near the VB edge when the BiVO_4_(010) surface is exposed to water. In order to identify the structural moieties involved, we carried out first-principles calculations based on DFT. Guided by our experimental measurements, we explicitly considered configurations involving molecular or dissociated water and the effect of electron doping, which imitates the n-type defects found in our samples, including oxygen vacancies. Our computational results show that the enhanced signal observed in the AP-ResPES measurements arises from small electron polaron formation. We find that while the adsorption of molecular water readily occurs on the pristine and undoped surface, it does not occur when excess charge is present at the surface. The main finding of our investigations is that the dissociation of water does occur on the BiVO_4_(010) surface, and the adsorbed hydroxyls can further stabilize the surface electron polarons. Our study represents an important contribution to the fundamental understanding of the electronic, structural, and chemical properties of the BiVO_4_/water interface based on a strategy combining measurements on single-crystalline samples and first-principles calculations. Our findings highlight the importance of surface defects in altering the surface reconstruction of ternary oxide surfaces in the presence of water.

## 2 Methodology

### 2.1 Experimental Methodology

We highlight here the main aspects of our experimental methodology. Further details may be found in ref (8) and in the Supporting Information.

#### 2.1.1 Sample Preparation

We intentionally doped our single-crystal BiVO_4_ with nominally 1 at. % Mo to improve the sample conductivity for our photoemission measurements. The Mo-doped BiVO_4_ single crystals were grown from Bi_2_O_3_, V_2_O_5_, and MoO_3_ (Aldrich, purity ≥ 99.99%) in air using the Czochralski technique with RF induction heating and automatic diameter control. Approximately 5 × 5 × 5 mm^3^ oriented pieces were first cut from the bulk Mo-doped BiVO_4_ crystal and then cleaved along the (010) plane. After introducing the cleaved crystals into the vacuum chamber, they were cleaned by heating to 300–320 °C in an O_2_(g) atmosphere. As reported previously,^8^ X-ray photoelectron spectroscopy (XPS) and low-energy electron diffraction analysis show that this reproducibly produces a clean and well-ordered surface with no indication of Mo surface segregation or carbon contamination on the surface at r.t. and under ultra-high vacuum (UHV). When transitioning from UHV to the experimental conditions of elevated water vapor pressure, a slight increase in carbon contamination on the surface was observed (see the Supporting Information). This is most likely due to the displacement of carbon-containing species from the analysis chamber walls upon water dosing. The carbon coverage (θ_C_) was estimated from the integrated peak areas of the C 1s and Bi 4f core-level (Figure S1a) spectra taken at a water pressure of 0.05 Torr and at a photon energy (PE) of 517.4 eV (i.e., in resonance with the V L_3_ 2p_3/2_ → 3d electronic transition, see below). From this analysis and using the “simulation of electron spectra for surface analysis” software (SESSA),^52^ θ_C_ was estimated to be equal to 0.03 ML (see the Supporting Information for the detailed description of the quantification). The normalized C 1s core-level peaks shown in Figure S1b, taken during the AP-ResPES experiment, show that the amount of carbon contamination and its chemical composition were stable throughout the measurement; that is, once 0.05 Torr of water pressure was reached, there was no further accumulation of carbon contamination on the surface. Note that the surface coverages reported, given in units of MLs, are expressed in terms of one BiVO_4_ ML (the *d*-spacing of one BiVO_4_ ML was taken as half a unit cell along the *b* direction, i.e., *d* = 5.76 Å).^8^

#### 2.1.2 Soft X-ray Photoelectron and Resonant Photoelectron Spectroscopies

The end station of beamline 11.0.2 at the Advanced Light Source (Lawrence Berkeley National Laboratory, Berkeley, USA) was used for AP-XPS and AP-ResPES measurements.^46,53^ The AP-XPS data were acquired using a photoelectron kinetic energy (KE) of 200 eV, a step size of 0.05 eV, and a pass energy of 20 eV for all core levels. Under these conditions, the total resolution (beamline plus electron spectrometer) was better than 100 meV at 735 eV at r.t.. AP-ResPES measurements were conducted by acquiring the VB spectra as the PE was scanned across the V L_3_ edge. The PE was scanned in steps of 0.1 eV. The VB spectra were acquired with a photoelectron KE step of 0.05 eV and an integration time of 0.3 s. Details on the procedure for spectral calibration and data analysis may be found in ref (8) and in the Supporting Information.

### 2.2 Computational Methodology

#### 2.2.1 Calculation Parameters

We additionally performed first-principles calculations based on DFT and the Kohn–Sham framework.^54,55^ Our computational methodology is built upon that of our previous work^37^ and we highlight the major aspects here. We carried out spin-polarized calculations using the Quantum ESPRESSO code^56,57^ and the norm-conserving pseudopotentials^58^ with a 90 Ry energy cutoff. The 6p^3^6s^2^5d^10^, 3d^3^4s^2^3p^6^3s^2^, and 2p^4^2s^2^ electrons were treated as the valence states for Bi, V, and O, respectively. We additionally used DFT + *U*(59,60) with *U*_eff_ = (*U* – *J*) = 2.7 eV applied to the vanadium 3d states.^61^ We previously found this approximation to be robust in capturing the localization properties of BiVO_4_.^6,37,62^ Symmetric slabs^63,64^ consisting of a 2 × 2 × 2 supercell of the bulk 24-atom *I*2/*b* cell were generated. Each slab had a minimum 20 Å of vacuum and a minimum of eight atomic layers. We use the *C*2/*c* cell convention when referring to the exposed (010) surface.

#### 2.2.2 Enumeration of Configurations

To build our computational models, we analyzed the XPS measurements of the Mo:BiVO_4_(010) surface exposed to water in order to identify the relevant species formed. From these measurements, we found that at around 0.01 Torr, the surface begins to hydroxylate. Both molecular water and dissociated water species; that is, hydroxyl groups, were adsorbed on the surface. An increased coverage of the adsorbed water species coincided with an increased amount of reduced vanadium sites (V^5+^ → V^4+^) at the surface. This inspired us to closely examine the relationship between the reduced vanadium sites and the nature of the adsorbed water, as further discussed below. Thus, we enumerated possible configurations and performed calculations to understand the separate and combined impacts of reduced vanadium sites and adsorbed water species on the electronic structure. In order to imitate the n-type conditions of our Mo:BiVO_4_ samples, we induced the localization of an excess electron on a surface V site (see the Supporting Information for further details). We note that other n-type defects such as oxygen vacancies^37^ are also possible sources of excess electrons in our Mo:BiVO_4_ sample and in thin BiVO_4_ films as found in devices.

**Figure 1:**
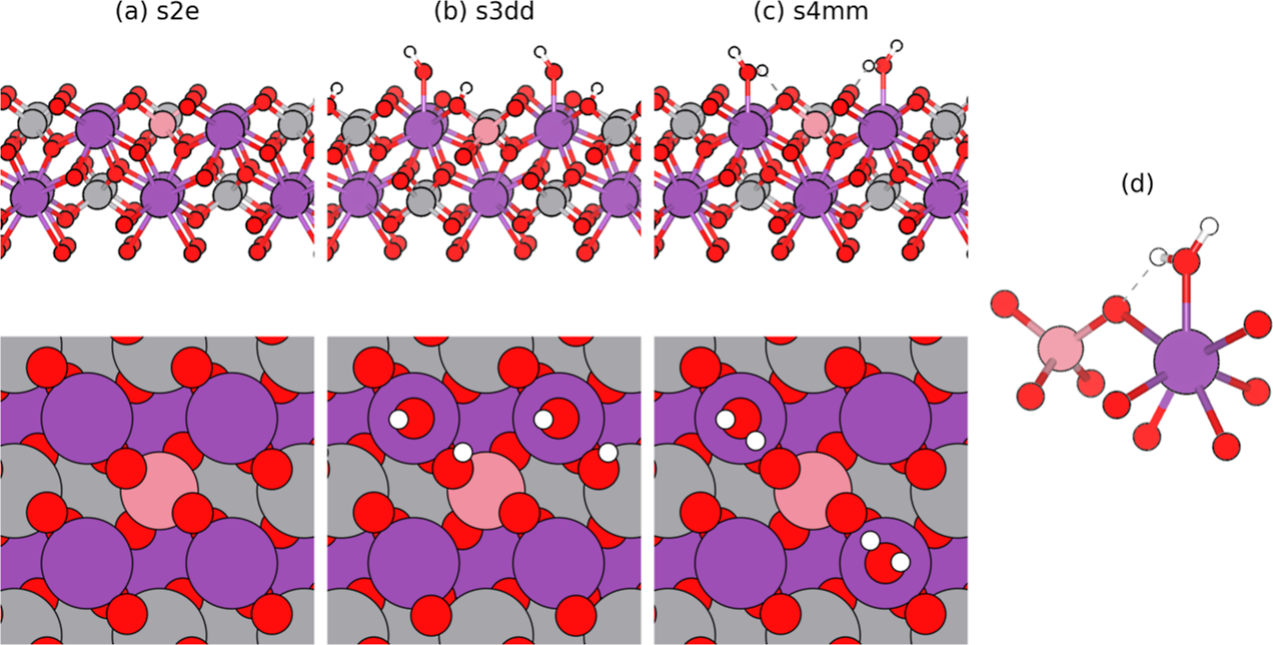
Selected models to illustrate the main structural configurations considered in our calculations: (a) pristine surface slab with surface polaron (s2e), (b) single-hydroxylated surface polaron with dissociated water molecules (s3dd), and (c) surface polaron with two water molecules oriented toward a particular VO_4_ (s4mm). Bi atoms are shown in purple, V atoms in gray, O atoms in red, and H atoms in white; the pink V atom denotes the site at which an electron polaron is initialized to form (see the main text). The local coordination environment of Bi (7-fold coordinated with the adsorbed water) and V (4-fold coordinated) of the main structural moiety in this study is shown in (d). An illustration of all major structural configuration variants may be found in Figure S2.

We differentiate between whether a computational sample has excess electrons, whether there is water adsorbed on the surface, and which orientation the adsorbed water species have relative to a chosen VO_4_ tetrahedron, and we enumerate our calculations across all representative combinations. In particular, we distinguish between whether a surface VO_4_ tetrahedron is coordinated with one or two hydrogen atoms from adsorbed water (see Figure 1). The relevant configurations discussed in the main text and their naming conventions are presented in Table 1. A complete table describing our notation (see Table S1) and further calculation details may be found in the Supporting Information.

**Table 1:** Summary of the Main Configurations Considered in Our Calculations^a^

label	added excess electrons	no. of water molecules at the surface	no. of water molecules oriented toward a single VO_4_
s3dd	yes	2 (dissociated)	1
s4dd	yes	2 (dissociated)	2
s3mm	yes	2 (molecular)	1
s4mm	yes	2 (molecular)	2
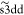	no	2 (dissociated)	1
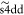	no	0 (dissociated)	2

a A complete list of configurations may be found in Table S1 of the Supporting Information. “s3” denotes a configuration where only one water molecule is oriented toward a selected VO_4_ tetrahedron, “s4” indicates that two water molecules are oriented toward a selected VO_4_ tetrahedron, and a tilde indicates that no electron doping was included. Appended to each “s#” is a string consisting of “m” and/or “d”, in which each characteristic represents the molecular or dissociated water adsorbed on each exposed surface of the slab, respectively.

We note that the configurations tested here for the adsorbed water were not exhaustive and did not include the possible influence of the nearby water; the latter will be a future topic of more detailed study. Nevertheless, the configurations enumerated above offer valuable insights into the possible conformations of the adsorbed species on the BiVO_4_(010) surface.

## 3 Results

### 3.1 AP-XPS Investigation of the Mo:BiVO_4_(010) Surface

The Mo:BiVO_4_(010) surface was investigated with a combination of soft X-ray ambient pressure (AP) photoelectron spectroscopy (AP-XPS) and AP-ResPES. XPS in UHV and AP-XPS were performed to determine the changes in the surface chemical composition and the oxidation states of Bi, V, and Mo for the pristine surface (UHV, ∼10^–9^ Torr) and upon exposure to 0.05 Torr of water (H_2_O) at r.t. (∼298 K).

**Figure 2:**
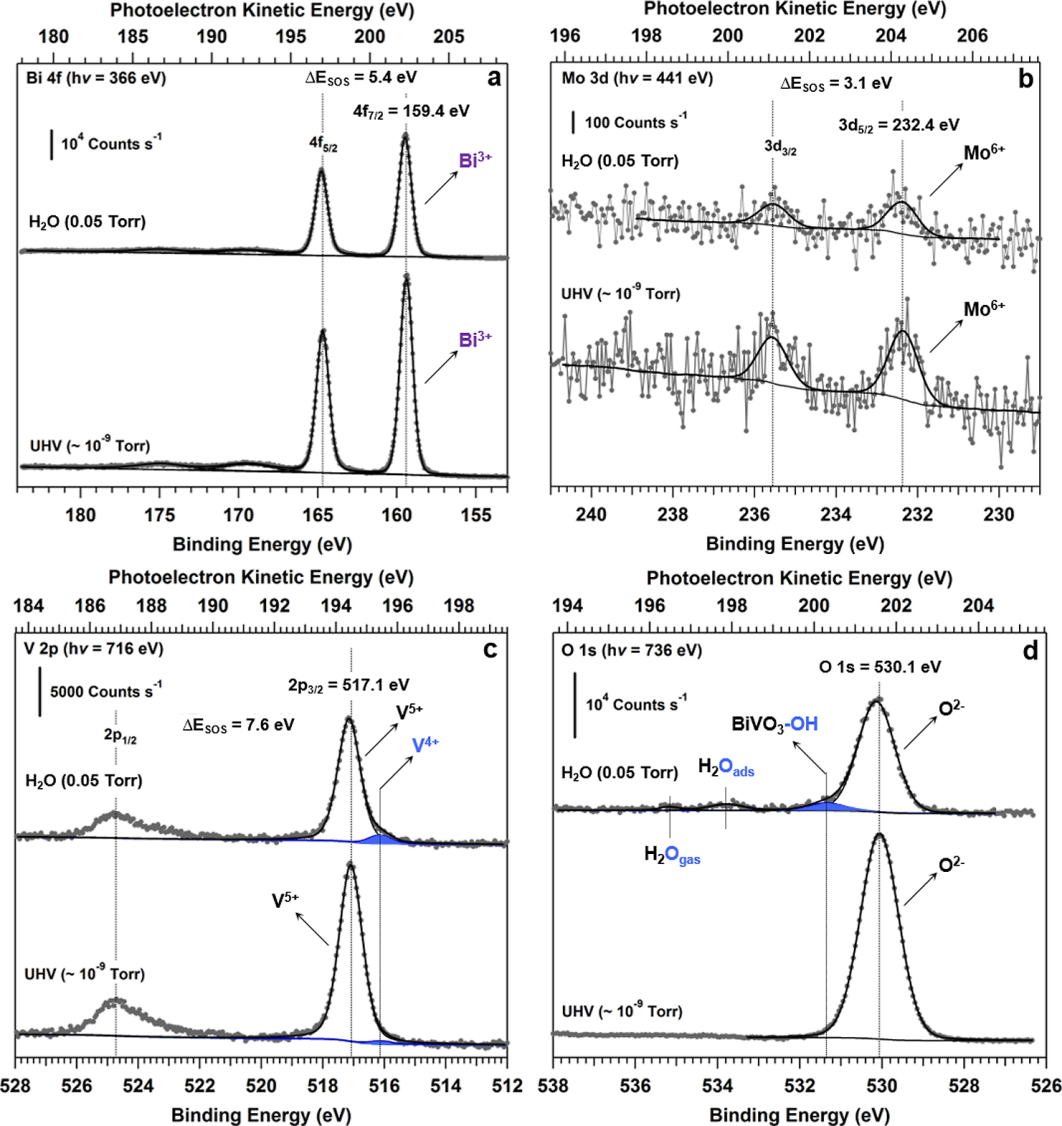
XPS investigation of Mo:BiVO_4_(010). The measurements were performed at r.t. on the pristine surface (UHV) and during exposure to 0.05 Torr of H_2_O (AP conditions). The PE used for each core-level spectrum was changed in order to provide the same photoelectron KE (∼200 eV) and therefore the same probed depth for all core-level spectra The inelastic mean free path of 200 eV photoelectrons in BiVO_4_ is equal to ∼8.3 Å (see the Experimental Methodology section for further details). The spin–orbit splitting, Δ*E*_SOS_, is the energy separation of the spectral features corresponding to the *S* = +1/2 and *S* = −1/2 states in the *J* = *L* + *S* spin–orbit split states. (a) Bi 4f (*h*ν = 366 eV), (b) Mo 3d (*h*ν = 441 eV), (c) V 2p (*h*ν = 716 eV), and (d) O 1s (*h*ν = 736 eV).

We first turn to XPS measurements in order to build a microscopic model for the interaction of the BiVO_4_(010) surface with water. For both UHV XPS and AP-XPS conditions, the core levels shown in Figure 2 were acquired by changing the PE to provide the same photoelectron KE and therefore the same probed depth for each core level.^52^ The photoelectron KE chosen was 200 eV, yielding an inelastic mean free path λ_e_ of 8.3 Å in BiVO_4_.^52^

In UHV conditions, we find that the binding energies (BEs) of Bi 4f_7/2_ (Figure 2a, 159.4 eV) and Mo 3d_5/2_ (Figure 2b, 232.4 eV) are consistent with the oxidation states of Bi^3+^ (6s^2^ configuration) and Mo^6+^ (4d^0^ configuration), respectively.^65−67^ As we have recently shown with near-edge X-ray absorption fine structure spectroscopy (NEXAFS),^8^ Mo^6+^ has a tetrahedral coordination environment, which suggests substitutional doping into V^5+^ sites. The excess electron introduced by the Mo^6+^ cations occupies a localized V 3d state. This finding is confirmed by the presence of a low BE component (BE = 516.2 eV, shaded in blue in Figure 2c) in the V 2p spectral region, which can be attributed to reduced surface vanadium (V^4+^).^38,39,68−70^ In UHV, the O 1s spectral region (Figure 2d) shows a single peak centered at a BE of 530.1 eV in line with previous assignments to O^2–^ reticular oxygen.^38,39,66,68−70^

Transitioning from the pristine Mo:BiVO_4_(010) surface in UHV to 0.05 Torr H_2_O vapor pressure, the Bi 4f and Mo 3d core levels do not show any significant changes. Under the same conditions, the V 2p spectrum (Figure 2c) is still dominated by the photoelectron peak centered at BE = 517.1 eV and attributable to V^5+^ (3d^0^ configuration),^38,39,66,68−70^ but the low BE shoulder assigned to V^4+^ increases in intensity, indicating that the exposure of the Mo:BiVO_4_(010) surface to water vapor has increased the amount of V^4+^ on the surface. This is accompanied by two new spectral components in the O 1s spectrum (Figure 2d), which are attributable to adsorbed −OH (BE = 531.4 eV) and H_2_O (H_2_O_ads_, BE = 533.8 eV).^46,66,71^ The presence of −OH component implies that water has dissociatively adsorbed on the Mo:BiVO_4_(010) surface.

This experimental finding, however, is in contrast to previous DFT calculations performed on undoped BiVO_4_ by Yang et al.^42^ on (010) and (011) surfaces and by MD simulations carried out by Oshikiri and Boero^40^ on the (100) surface. In both of these works, the adsorption of dissociated water molecules was not observed, but rather only the adsorption of molecular water at Bi surface sites via a Bi–O interaction (see e.g., Figure 1d) was reported. From our experimental results, we can determine the surface coverage (θ) of the adsorbed −OH (θ_–OH_) and the corresponding coverage of reduced vanadium at the surface ().^72^ At 0.05 Torr of H_2_O,  = 0.08 ML. Note that the spectral fingerprint of oxygenated carbon species in the O 1s core-level spectrum overlaps with that of the adsorbed −OH. This contamination accounts for about 0.02 ML of the observed adsorbed oxygen-containing species in this BE range (see the Experimental Methodology section and the Supporting Information for spectra and carbon contamination coverage calculations) and leads to an estimated hydroxyl coverage of θ_–OH_ = 0.07 ML. This leads to a coverage ratio of hydroxyl to reduced vanadium of ∼0.9 (i.e.,  ∼ 0.9). If one dissociated water molecule, which can form two adsorbed surface hydroxyl groups, led to one reduced vanadium in the surface, this ratio should be 2. Our ratio of 0.9 suggests an excess amount of reduced vanadium on the surface upon its hydroxylation. Below, we propose that the reduced vanadium is the result of hydroxyl-induced localization of the excess electrons that already exist in the sample from the Mo dopants. However, increasing the water vapor pressure leads to surface hydroxylation and a corresponding reduction of V^5+^ to V^4+^, and these processes are localized to the surface region of BiVO_4_. Based on these observations, we can conclude that H_2_O indeed dissociates at the Mo:BiVO_4_(010) surface, and in the following, we seek to identify a microscopic model to interpret these observations. In particular, we investigate what structural moieties result from water dissociation and surface hydroxylation using DFT calculations, identify which of these structural moieties stabilize excess electrons at the Mo:BiVO_4_(010) surface, and discuss the possible reasons why water dissociation has not been observed in previous computational studies.

### 3.2 AP-ResPES Investigation of the Mo:BiVO_4_(010) Surface

**Figure 3:**
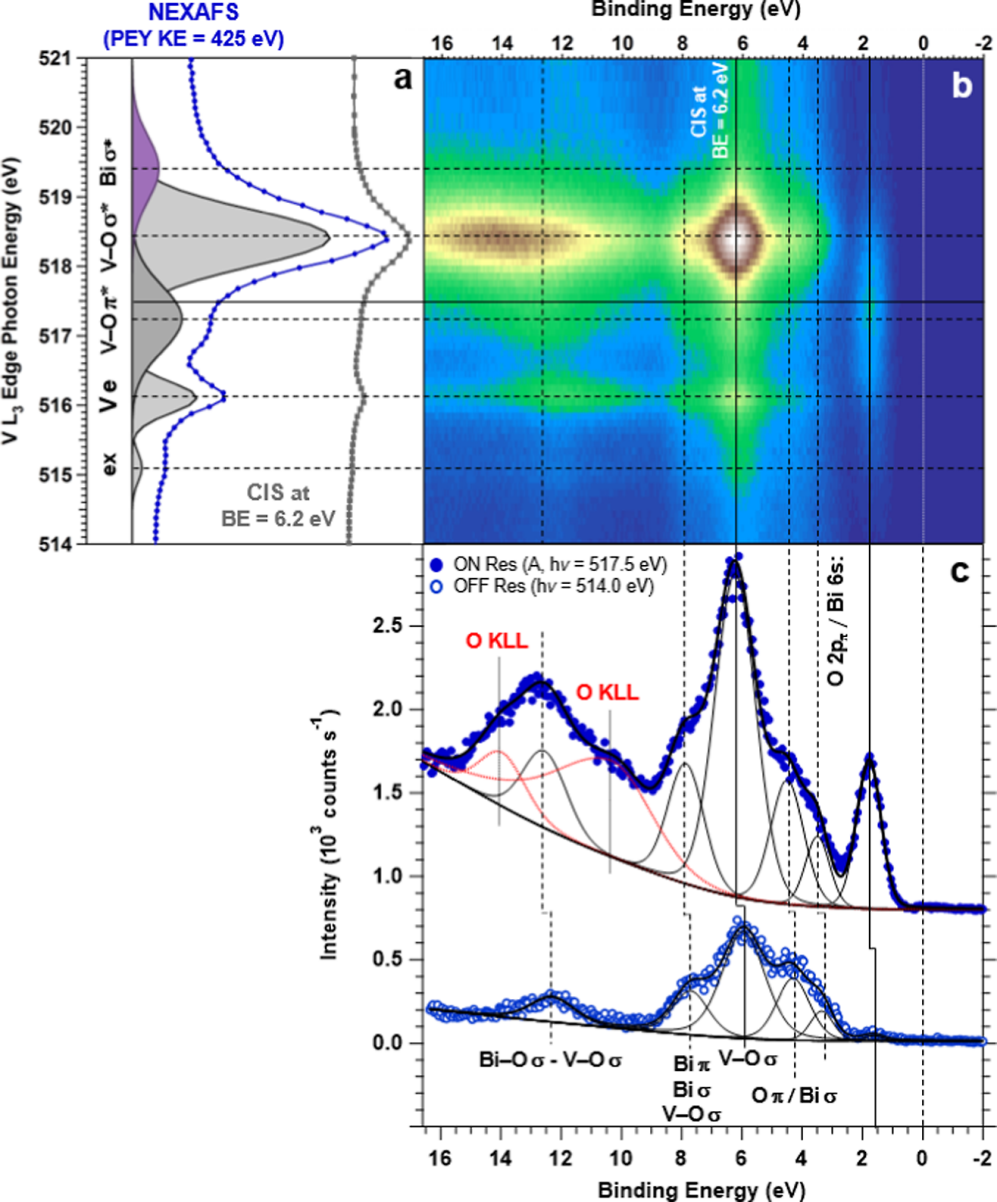
AP-ResPES of the Mo:BiVO_4_(010) surface at r.t. and a water pressure of 0.05 Torr. (a) The PE was scanned across the V L_3_ edge inducing, within the dipolar approximation, the 2p_3/2_ → 3d optical transition. The blue curve is the NEXAFS spectrum obtained in a partial electron yield (PEY) at a KE of 425 eV (see the Experimental Methodology details and the Supporting Information for further details), whereas the gray profile is the CIS profile obtained by slicing the 2D map reported in (b) at a BE of 6.2 eV, corresponding to the intensity maximum. The overlap of the two spectra confirms that the features in the VB undergoing the intensity enhancement under resonant conditions are related to V d states. (b) 2D map obtained by plotting the VB spectra as a function of PE. In (c), the comparison between the VB spectra taken in off- and on-resonance conditions is shown. Note that the O KLL Auger transitions reported as dotted red lines are due to the concomitant direct O Auger emission as the PE approaches the O KLL KE (NEXAFS, PEY, BE, CIS, VB, and PE).

Surface hydroxylation^46,71^ not only changes the chemical composition of the surface but can also modify its electronic properties.^20^ To investigate the changes in the valence states of the Mo:BiVO_4_(010) surface upon hydroxylation, we performed AP-ResPES^66^ in a water ambient of 0.05 Torr and at r.t.. ResPES is a powerful tool to resolve the various elemental contributions to the VB structure of materials.^73−76^ Element specificity is obtained by exciting the electrons of the VB using photons with energies (PE) near the absorption/ionization edges of the selected element. The enhancement of the photoelectron signal intensity arises from the constructive interference of two different interaction channels: (i) electrons directly photoemitted from the VB and (ii) electrons emitted at the same KE through a resonant absorption-initiated Auger decay process.^77,78^ This intensity enhancement makes it possible to probe weak VB features whose detection via direct VB photoemission can be difficult. In this study, we monitor the VB spectrum as the PE is scanned across the V L_3_ edge corresponding to the 2p_3/2_ → 3d electronic transition in the dipolar approximation (see the corresponding NEXAFS spectrum in Figure 3a). Figure 3b shows the VB spectra as a function of PE as it is scanned across the V L_3_ absorption edge in the form of a 2D map. The gray curve reported in Figure 3a is the constant initial state (CIS) profile obtained by slicing the 2D map reported in Figure 3b at a BE of 6.2 eV, corresponding to the maximum intensity enhancement in the VB spectrum. The overlap of the NEXAFS and CIS spectra confirms that the features in the VB undergoing the intensity enhancement under resonant conditions are related to V d states. In Figure 3c, a comparison of the VB spectra taken off and on resonance is shown; the comparison highlights the increase in intensity and the change in the spectral shape induced by the resonance excitation conditions.

**Figure 4:**
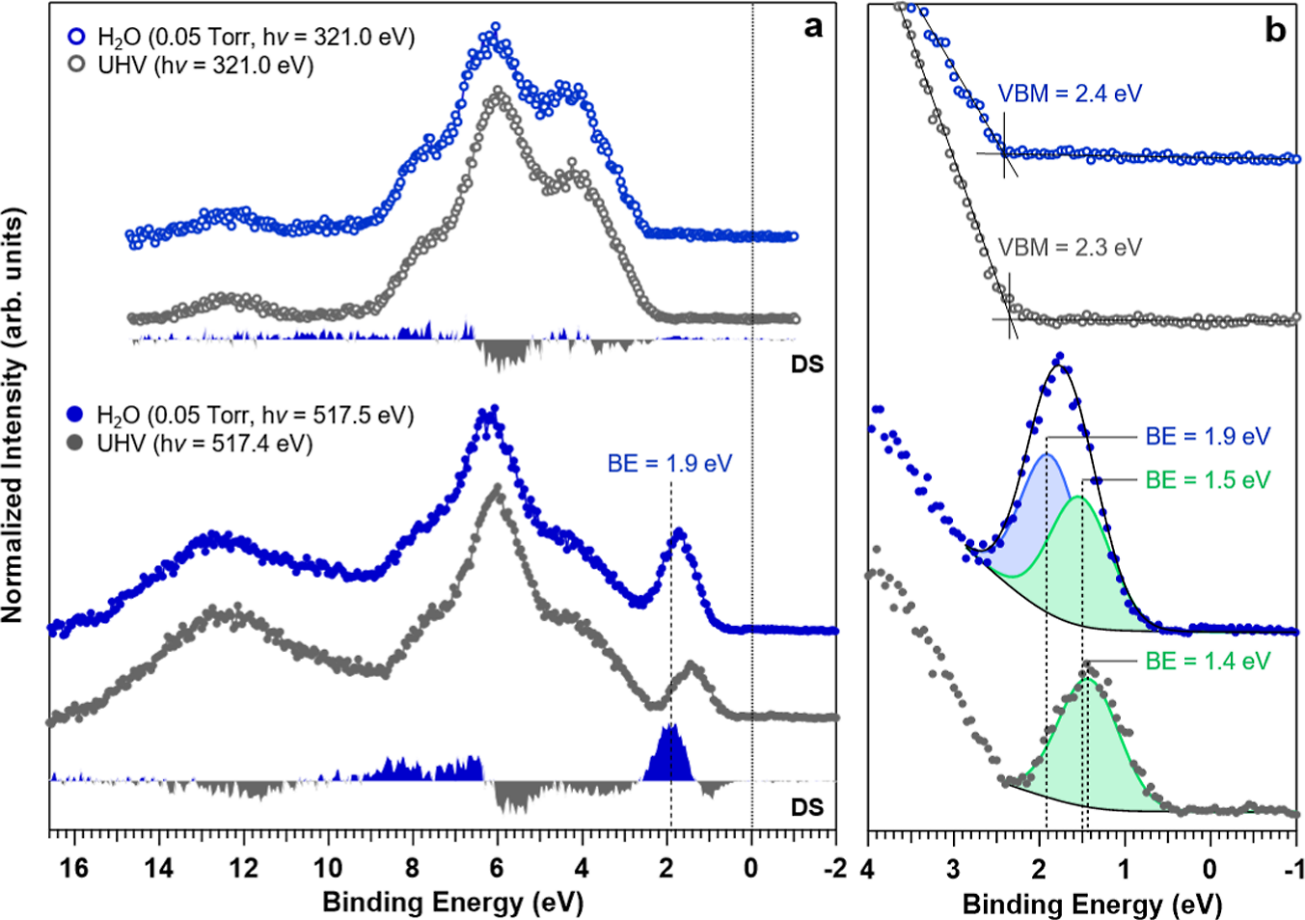
(a) VB spectra in off- (top) and on-resonance (bottom) conditions for the clean (UHV) and Mo:BiVO_4_(010) surface in 0.05 Torr H_2_O and (b) magnification and deconvolution of the VB spectrum in the BE range between −1 and 4 eV, emphasizing the electronic structure at the upper edge of the VB (VBM, UHV, DS: difference spectrum).

As reported in our previous work^8^ and in the comparison shown in Figure 4a, the V–O σ feature located at about 6 eV in the VB spectrum undergoes a significant increase when changing from off-resonance to on-resonance excitation, both on the clean and hydroxylated Mo:BiVO_4_(010) surfaces. This is in line with the partial density of states obtained from DFT calculations,^79−81^ which show that the middle of the VB is dominated by hybridized V 3d and O 2p states. Furthermore, for the clean Mo:BiVO_4_(010) surface measured in UHV, we observed a spectral feature above the VB maximum (VBM) within the energy band gap at a BE equal to 1.4 eV. A PE of 517.4 eV yielded the maximum intensity of this feature as previously observed.^8^ The nature of the observed resonantly enhanced feature was assigned to highly localized, reduced V^4+^ moieties formed by excess electron localization in V 3d-derived orbitals, where the excess electrons are provided by the Mo dopants. This feature is associated with the formation of a defect state in the energy band gap and corresponds to the formation of a small polaron at a VO_4_ tetrahedral site.

Upon water adsorption on and hydroxylation of the Mo:BiVO_4_(010) surface, three main observations can be made:

1. The intensity of the resonantly enhanced feature above the VBM increases compared to the surface measured in UHV conditions.
2. The maximum intensity of the resonant state occurs at the same PE (within experimental uncertainty) as for the surface in UHV conditions.
3. The position of the resonantly enhanced above VBM feature shifts to higher BE values compared to its position in UHV.

Together, these findings suggest that the resonant state arises from the same set of V 3d (localized) orbitals irrespective of whether the surface is pristine or exposed to water. A detailed analysis of the resonant state for the surface in 0.05 Torr H_2_O allows the disentangling of its two spectral components, separated by 0.4 eV (Figure 4b). The low BE component (BE = 1.5 eV) represents the resonant contribution from the reduced V^4+^ generated by the localization of the excess charge from the Mo^6+^ dopants or more generally non-hydroxylated VO_4_ moieties, as previously assigned;^8^ the higher BE component (BE = 1.9 eV) is related to the conversion of surface V^5+^ to V^4+^ upon the adsorption of water and likely the hydroxylation of the VO_4_ moieties. With this additional insight, we now turn to DFT calculations to correlate the structural moieties of the adsorbed water species with their corresponding electronic structures and to the spectral features observed experimentally.

### 3.3 Structural Motifs and Stability of Water Adsorption on the (010) Surface

The electronic and atomic structures of the bare BiVO_4_(010) surface with and without oxygen vacancies under vacuum were reported in our previous study.^37^ Here, we explore different configurations, modeling the presence and absence of defects via electron doping to understand what moieties of adsorbed water may lead to the formation of surface polarons, which in turn could explain the enhanced defect state observed in our AP-XPS and AP-ResPES measurements.

Our AP-XPS and AP-ResPES measurements support and inform our computational models. The model we first consider is that of water dissociating and leading to the binding of an OH group to the Bi sites and of a proton to a neighboring O^2–^ site of the VO_4_ moiety. The substitution of Mo^6+^ on V^5+^ suggests that an excess electron from Mo is donated to the host lattice to form V^4+^. Likewise, the two electrons from an oxygen vacancy lead to the formation of V^4+^.^37^ It has been shown in numerous computational studies that small electron polarons have a V 3d characteristic and form in the presence of oxygen vacancies^37,62^ and/or Mo-doping^11^ (see also Figure S4 for density of states). Thus, we model our Mo:BiVO_4_(010) single-crystalline samples as slabs with excess electrons localized on V sites.

We first considered the adsorption of molecular water in the absence of electron doping ( and  configurations). In general, the adsorption of molecular water is energetically favorable (by around 0.5 eV for the configurations calculated here) compared to the energy of the separate BiVO_4_ surface and water molecules, consistent with previous DFT studies.^40,43^ However, upon adsorption of molecular water, no in-gap defect state was observed, indicating that the adsorption of molecular water cannot explain the enhanced intensity of the spectral feature above the VBM observed in AP-ResPES measurements. When electron doping was introduced into the system, we did not find electronically stable configurations with the adsorption of one (s3mm) or two (s4mm) water molecules, suggesting that molecular water adsorption in the presence of surface excess charge is unfavorable.

Next, we turned to electronically doped configurations (s3dd and s4dd) involving the adsorption of two dissociated water molecules. We found that both these configurations had a lower energy than that of the sum of the bare BiVO_4_(010) surface with a surface polaron and the energy of an equivalent number of isolated water molecules. This suggests that the adsorption of dissociated water molecules stabilizes a surface polaron, leading to an increased concentration of localized charge at the surface. We also found that the double-hydroxylated configuration was more stable than the single-hydroxylated (s3dd) configuration by around 295 meV. Additionally, we considered the case of the adsorption of dissociated water molecules without electron doping. We could stabilize and relax the single-hydroxylated configuration (s3dd) but not the double-hydroxylated configuration (s4dd). This suggests that while the adsorption of dissociated water molecules is possible in the absence of surface excess charge, there are a limited number of stable structural moieties. Mixed configurations with both dissociated and molecular adsorbed water were also considered. Further details of these and other configurations are discussed in the Supporting Information.

### 3.4 Electronic Structure and Localization Properties of Water Adsorbed on the Electronically Doped BiVO_4_(010) Surface

**Figure 5:**
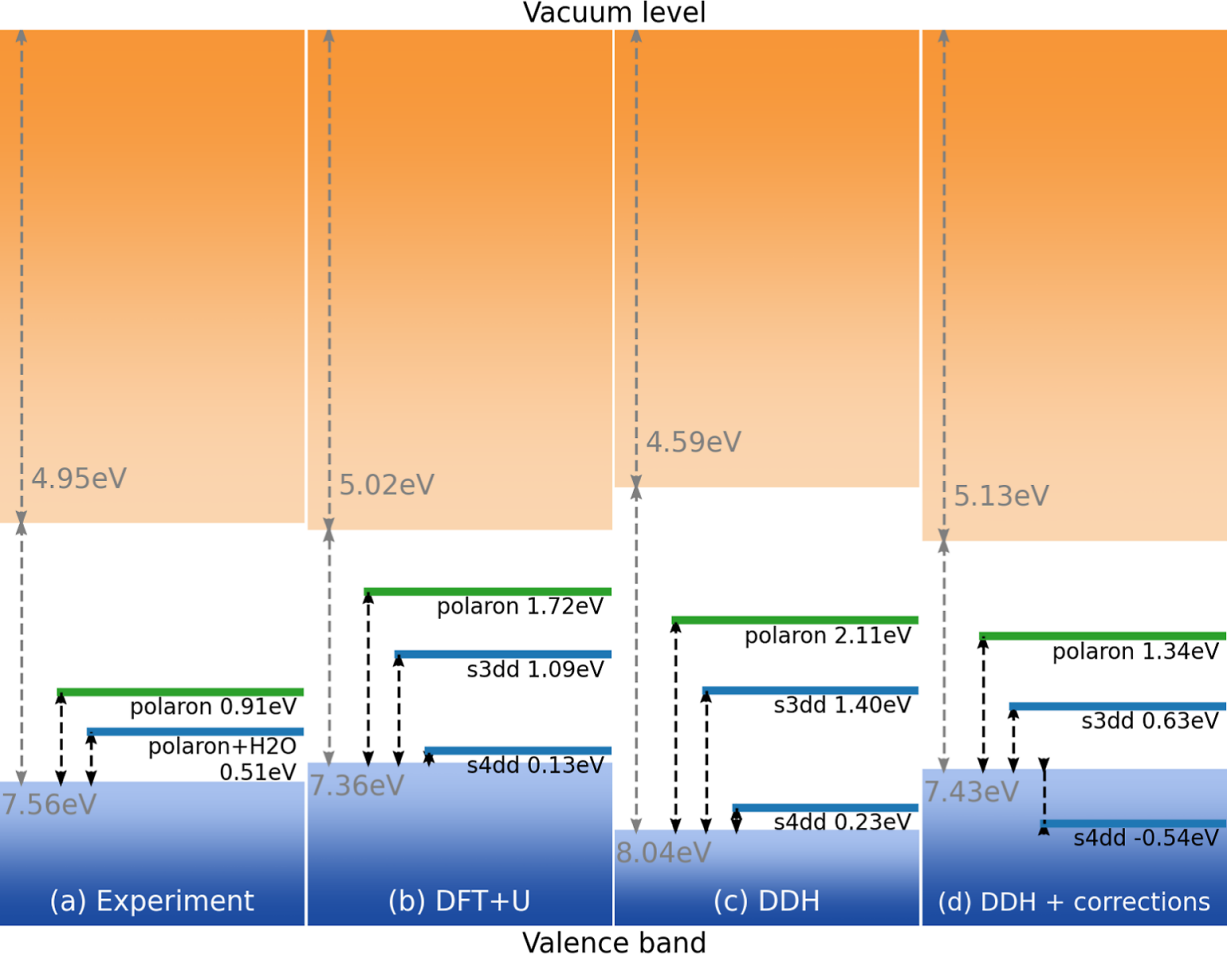
Relative positions of defect states with respect to the VBM as identified in (a) measured samples in 0.05 Torr H_2_O based on the analysis of AP-ResPES spectra in Figure 4 and calculations using (b) DFT + *U*, (c) one-shot calculations using the dielectric-dependent hybrid functional (DDH), and (d) one-shot DDH calculations with corrections for temperature renormalization, nuclear quantum effects, spin–orbit coupling, and exciton contributions based on the path-integral MD simulations from ref (83). The experimental values of the band edges in (a) are derived from the VBM vs *E*_Vacuum_ on the clean surface reported in ref (8) and shifted by +0.1 eV to take into account the measured BE shift of the VBM when transitioning from UHV to 0.05 Torr of H_2_O (see Figure 4 and text for further details). Green lines correspond to configurations with defects/charge doping but no hydroxylation, and blue lines correspond to configurations with defects/charge doping and hydroxylation. The s3dd and s4dd configurations correspond to a surface polaron localized on a VO_4_ tetrahedron with adsorbed dissociated water molecules involving single and double hydroxylation, respectively.

For the configurations we could stabilize in our calculations, we investigated the electronic structure, focusing on the relative position of the defect level introduced by the adsorbed species, with respect to the VBM, as shown in Figure 5. The experimental values of the band edges reported in Figure 5a are derived from the procedure outlined in our previous work.^8^ The reference energy was shifted from the Fermi level to the vacuum level according to the following relation: *E* = −(BE + φ). The work function φ, equal to 5.15 ± 0.05 eV, was determined by measuring the secondary electron cutoff on the clean Mo:BiVO_4_(010) surface under vacuum.^8^ The VBM for the hydroxylated surface, measured at a BE of 2.4 eV (Figure 4b), is therefore shifted to 7.5 eV below the *E*_vacuum_ in Figure 5a. As provided by previous DFT calculations conducted on the clean BiVO_4_(010) surface,^40,79,82^ the conduction band minimum (CBm) is placed 0.3 eV above the Fermi level.^8^ Hence, using the same equation reported above, we can place the CBm at 4.85 eV below the *E*_Vacuum_. On the other hand, we observe a BE shift of +0.1 eV of the VBM position upon surface hydroxylation (Figure 4b). Under the assumption that this is a rigid energy shift, we corrected the CBm energy as well, thereby placing the CBm at 4.95 eV below the *E*_Vacuum_. We note that the calculated band alignment and Fermi level of the undoped, clean BiVO_4_(010) surface^37^ are in good agreement with measurements based on single-crystalline samples in UHV.^8^ We computed the position of the polaron defect level at the Brillouin zone center. As discussed in the Supporting Information, we estimate an error bar of about 0.1 eV in determining the relative position of the defect level with respect to the band edges. Table S2 presents additional data on how the band gap and polaron defect level positions vary with hydroxylation.

In order to determine which configuration’s electronic structure could represent the enhanced small polaron signal detected in our AP-ResPES measurements upon water adsorption and dissociation, we compare the relative position of the polaron levels for those configurations exhibiting an inter-band electronic level, that is, the single- and double-hydroxlyated configurations with a surface polaron (s3dd and s4dd). The polaron levels are aligned to the absolute positions of the band edges for the BiVO_4_(010) surface. We directly compare the results obtained for experimental samples in UHV conditions with those from calculations on the unhydroxylated electron-doped BiVO_4_(010) surface; we also compare the results obtained for our experimental samples in 0.05 Torr H_2_O conditions with those from calculations on the single- and double-hydroxylated (s3dd and s4dd) configurations. Figure 5 shows the experimental data (Figure 5a) compared to computational results obtained with various functionals (Figure 5b–d). In Figure 5d, additional corrections mainly based on finite-temperature effects are included, which essentially renormalize the band edges. Additional details on these corrections may be found in our previous study^37^ and are discussed further in the Supporting Information. For the unhydroxylated BiVO_4_(010) surface with electron doping (s2e, green line in Figure 5b), the defect level of the surface V polaron is 1.72 eV above the VBM (0.34 eV below the Fermi level). For the electron-doped single-hydroxylated configuration with dissociated water (s3dd), the defect state is 1.09 eV above the VBM (0.97 eV below the Fermi level). Comparing the results for configurations with no water molecules and surface polaron (s2e) with those with dissociated water molecules and surface polaron (s3dd) reveals that the presence of hydroxyl groups leads to a polaron defect state farther from the Fermi level, in agreement with the experimental findings. This finding suggests that the presence of dissociated water molecules energetically stabilizes the surface polaron on VO_4_, thereby implying that the exposure of the BiVO_4_ surface to water enhances the formation of electron polarons at the surface. The density of states for the s2e, s3dd, and s4dd configurations may be found in the Supporting Information. We also tested whether explicitly modeling ∼1% Mo-doping would lead to qualitatively different results by replacing two V atoms in the bulk-like region of the slab with Mo for the s3dd configuration. We found that the defect state in the band gap remained a deep defect state around 1.05 eV above the VBM.

## 4 Discussion

Our computational results reveal several insights that can be used to interpret our AP-XPS and AP-ResPES results. First, the adsorption of water molecules on the (010) surface can occur in the absence of the nearby excess electronic charge, explaining the observed peak assigned to the adsorbed molecular water in our AP-XPS spectra. Second, of the configurations tested, we find that the enhanced intensity of the above VBM feature observed in our AP-ResPES spectra may be explained by the presence of the adsorbed dissociated water. The adsorption of dissociated water leads to proton transfer to and hydroxylation of the surface VO_4_ moieties, as can be seen in the single-hydroxylated (s3dd) configuration, and coincides with the formation of a surface small polaron (i.e., nominal V^4+^ at the surface) as well as a polaron defect state in the band gap. The polaron defect state is stabilized by the hydroxyl groups relative to the state without dissociated water, as evidenced by its deeper (i.e., closer to the VB edge) polaron defect level. Thus, the increase in intensity and higher BE of the resonantly enhanced above VBM feature in AP-ResPES spectra may be attributed to the stabilization of small polarons due to the dissociation of adsorbed water and the formation of hydroxyl groups on VO_4_ tetrahedra. The stabilization of small polarons at the BiVO_4_(010) surface may be attributed to the structural distortions of the VO_4_ moiety when dissociated water adsorbs (see Table S3). While adsorption of molecular water barely distorts the structure of the VO_4_ tetrahedron, the adsorption of dissociated water leads to a larger structural distortion of the VO_4_ tetrahedron that helps to stabilize an electron polaron. These structural distortions would also be expected to occur in cases where other n-type defects such as oxygen vacancies are present. We note that our computational strategy for modeling the adsorption of water vapor is intended to reflect the conditions in our AP-ResPES experiments (pH_2_O = 0.05 Torr), thereby not accounting for interactions with neighboring water species that would be present at the semiconductor/liquid electrolyte interface. The study of the fully solvated surface and the interaction of adsorbed and dissociated water molecules with aqueous-phase species will be a topic of future work.

Our combined experimental and computational study provides a systematic understanding of the nature of water adsorption on the Mo:BiVO_4_(010) surface. Due to the synergy between the experiment and theory, we were able to construct meaningful structural models. Interestingly, previous computational studies had not reported or observed the dissociation of water on the BiVO_4_(010) surface. A few reasons could underlie this apparent inconsistency. First, in MD simulations, for systems in which dissociation does occur, a dissociation event may be a rare event and difficult to observe for short trajectories (∼5 ps long trajectories were used in refs (40) and (44)). Second, observing molecular or dissociated water species at the surface may also depend on whether the initial configuration contains dissociated or molecular water species. For instance, Guo and colleagues^22^ simulated trajectories up to 16 ps of various semiconductor/water interfaces and found that at the interface of anatase TiO_2_, water remained molecular if it was initialized as molecular and remained dissociated if it contained dissociated water. In ref (43), an electrostatic correction was used to correct for the presence of any dipole moments in their asymmetric slabs, which could have affected the interaction of water with the surface. Finally, many of the studies in literature studies^22,40,43^ considered only the pristine undoped (010) surface, which does not account for the impacts of n-type defects such as Mo (electron) doping and oxygen vacancies as we have done here.

While our calculations qualitatively corroborate our measurements, there are quantitative differences, particularly regarding the relative position of the polaron level obtained with DFT + *U*. We tested the robustness of our DFT + *U* calculations by turning to (one-shot) calculations carried out with the dielectric-dependent hybrid (DDH) functional^84^ for configurations with dissociated water species. We show in Figure 5b,c that the relative positions of the polaron defect level with and without adsorbed water does not qualitatively change when using DFT + *U* or hybrid functional calculations. We also considered renormalization effects of the energy band gap due to finite temperature, spin–orbit coupling, and nuclear quantum effects. Drawing on studies from Wiktor and colleagues,^37,44,85^ we estimate the correction to the band edges beyond DFT + *U*, which is shown in Figure 5d. These corrections improve the agreement with the experiment in the relative position of the polaron defect level with respect to the VBM. Overall, from the closest one-to-one comparison presented in this study (see Figure 5a,d), we find sufficiently good agreement between theory and experiments in the ordering and relative position of the polaron defect levels for the BiVO_4_(010) surface under UHV and with water exposure. This allows us to identify the enhanced polaron peak as arising from localization of surface charge at VO_4_ tetrahedral sites caused by the adsorption of dissociated water and hydroxylation of the surface.

Other factors that are important to consider include the limited number of configurations sampled here due to the limited size of our supercell. We note that our calculations have been conducted with a 0.5 ML coverage of water species, whereas experimentally, we found coverages of 0.074 ML. Surface coverage has been known to influence the adsorption energetics and configurations in other complex oxide surfaces such as TiO_2_.^19^ Lower surface coverages merit future computational investigation; however, we note that it is computationally expensive as it requires significantly larger simulation cells. We also anticipate the structural moieties present in the experiment to be more diverse and yield a wider energy range of positions in defect levels weighted by their energetic stability compared to those in our calculations. Additionally, our calculations included only adsorbed and dissociated water and not surrounding water, which may also influence the positions of the band edges and defect level, particularly for the explicit BiVO_4_/electrolyte interface.^44^ Work is under way on dynamical studies to better capture the influence of neighboring water species and sample a greater number of possible adsorption configurations.

## 5 Conclusions

In summary, we presented a combined experimental and computational study on identifying spectroscopic signatures and structural moieties for the adsorption and dissociation of water on single-crystalline Mo:BiVO_4_(010) surfaces. We elucidated conditions for which the adsorption of molecular and dissociated water species can occur. In particular, we highlighted the utility of resonant photoemission spectroscopy in ambient conditions (AP-ResPES) as a valuable tool for directly probing elemental and orbital contributions to the electronic states, which can be readily compared to DFT calculations. In this study, AP-ResPES was used to understand the electronic structure of electron polarons for Mo:BiVO_4_(010) surface in UHV and in 0.05 Torr water vapor pressure. By comparing AP-ResPES spectra for samples under UHV conditions and in 0.05 Torr H_2_O pressure, we observed that the adsorption of water leads to an enhanced intensity of the feature near the VBM when scanning the PE across the V L_3_ edge. We utilized first-principles calculations based on DFT + *U* and hybrid functionals to computationally investigate possible configurations of adsorbed water species that would cause this increase in intensity. This strategy allowed us to identify the structural moieties that are involved in the hydroxylation of the BiVO_4_(010) surface. We found that both molecular and dissociated water moieties may adsorb on the undoped (010) surface, consistent with our AP-XPS measurements. More interestingly, we found that excess electrons from n-type defects such as Mo are further stabilized by the presence of adsorbed dissociated water to form electron polarons localized on surface VO_4_ polyhedra. The additional stabilization of electron polarons from adsorbed dissociated water on the electronically doped BiVO_4_(010) surface explains the enhancement of the above VBM feature in our AP-ResPES measurements of Mo:BiVO_4_(010) in 0.05 Torr water vapor. We anticipate similarities in the presence of an in-gap electronic state and in the nature of hydroxylation to occur when other n-type defects such as oxygen vacancies are present, though further investigation is merited. In determining the hydroxylation species expected on the Mo:BiVO_4_(010) surface, our study paves the way for elucidating atomic-level mechanisms of water splitting reactions and for subsequently understanding the oxide semiconductor/electrolyte interface.

Overall, our strategy enables us to disentangle the contributions of defects to the spectroscopic signals associated with the exposure of the Mo:BiVO_4_ surface to water and to identify the relevant structural moieties for an oxide surface in contact with water. We demonstrated the utility of using AP-ResPES to probe the elemental contributions of the (surface) electronic structure under ambient conditions. Finally, we provided a combined experimental and computational framework for systematically understanding the nature of adsorbed species on complex oxide surfaces and their interactions with water.

## Supporting Information Available

Further details on experimental methods, calculation methods, and additional results of the calculated configurations (PDF)
