# Supplementary material for: Influence of Excess Charge on Water Adsorption on the BiVO4(010) Surface
Source: J Am Chem Soc. 2022 Sep 8;144(37):17173–85. doi: 10.1021/jacs.2c07501 (PMC9501793; doi:10.1021/jacs.2c07501)
Supplement: Supplementary file 1 — ja2c07501_si_001.pdf [file ja2c07501_si_001.pdf]

Supporting Information:
The influence of excess electrons on water adsorption on the $BiVO_4$ (010) surface

Wennie Wang[1*†], Marco Favaro[2*], Emily Chen[3], Lena Trotochaud[4‡], Hendrik Bluhm[4#], Kyoung-Shin Choi[5], Roel van de Krol[2,6], David E. Starr[2], Giulia Galli[1,3,7]

[1] *Pritzker School of Molecular Engineering, University of Chicago, Chicago, IL 60637, USA*
[2] *Institute for Solar Fuels, Helmholtz-Zentrum Berlin für Materialien und Energie GmbH, Hahn-Meitner-Platz 1, 14109, Berlin, Germany*
[3] *Department of Chemistry, University of Chicago, Chicago, IL 60615, USA*
[4] *Chemical Sciences Division, Lawrence Berkeley National Laboratory, Berkeley, CA 94720, USA*
[5] *Department of Chemistry, University of Wisconsin-Madison, Madison, WI 53706, USA*
[6] *Institut für Chemie, Technische Universität Berlin, Straße des 17. Juni 124, 10623 Berlin, Germany*
[7] *Materials Science Division and Center for Molecular Engineering, Argonne National Laboratory, Lemont, IL 60439, USA*

[*] equal contribution
[†] Current address: McKetta Department of Chemical Engineering, University of Texas at Austin, Austin, TX 78705 USA; wwwennie@che.utexas.edu
[‡] Current address: Center for Water, Sanitation, Hygiene, and Infectious Disease, Duke University, Durham, NC, 27701 USA
[#] Current address: Fritz-Haber-Institut der Max-Planck-Gesellschaft, Faradayweg 4-6, 14195 Berlin, Germany

E-mail: david.starr@helmholtz-berlin.de, gagalli@uchicago.edu

Table of Contents

Experimental Methods 2
Calculation Methods 6
Additional results of calculated configurations 9

Experimental Methods

Our experimental procedure follows our previous work on single crystalline $BiVO_4$ in ultra-high vacuum (UHV) conditions.[1] We highlight relevant aspects here.

Mo:$BiVO_4$ Crystal Growth

We intentionally doped our single crystal $BiVO_4$ with nominally 1 at% Mo in order to improve the sample conductivity and prevent sample charging during our photoelectron spectroscopy measurements. The Mo-doped $BiVO_4$ single crystals (hereafter Mo:$BiVO_4$) were prepared at the Leibniz Institut für Kristallzüchtung (IKZ, Berlin, Germany). The growth melt was prepared from the starting oxides Bi_2O_3, V_2O_5, and MoO_3 (Aldrich, purity ≥ 99.99%). The crystal was grown in air using the conventional Czochralski technique with RF-induction heating and automatic diameter control. Platinum crucibles (40 mm in diameter and height) were used as the melt container, due to the low melting point of $BiVO_4$ (1213 K). To adjust the required temperature gradients, an active afterheater (diameter: 40mm, height: 70 mm) was installed above the crucible. Thermal insulation of the afterheater was provided by an outer alumina ceramic tube filled with alumina granules. We obtained the seed crystal from spontaneously crystallized material. The pulling rate was 1.0 mm h^{-1} and the rotation rate was 10 rpm. After growth, the crystal was cooled down to room temperature (r.t., ~298 K).

Surface Preparation

Approximately 5 x 5 x 5 mm^3 samples of the Mo: $BiVO_4$ crystal were oriented and cut from the bulk crystal. The sample was first cleaved on the bench top parallel to the (010) plane. The sample was then thoroughly rinsed with absolute ethanol (Aldrich, > 99.99%) and Milli Q water (ρ =18.2 MΩ · cm) and dried with a N_2(g) stream. The sample underwent an annealing cycle in UHV conditions and then in an O_2(g) background (1 Torr). As reported in our previous work [1], XPS analysis revealed that this procedure reproducibly produces a clean, and well-ordered surface with no indication of Mo surface segregation. The absence of carbon contamination on the surface was verified by the lack of a signal in the C 1s core level range (binding energy, BE, 280-295 eV) upon returning to room temperature and UHV conditions. Transitioning from UHV to AP conditions, a slight increase in carbon contamination was observed, likely from the displacement of carbon containing species from the analysis chamber walls.

The corresponding carbon coverage (θ_C) was estimated as follows. First, upon background subtraction, we obtained the area of the C 1s and Bi 4f core level peaks (**Figure S1a**) taken at a water pressure of 0.05 Torr and at the same photon energy (PE) of 517.4 eV (i.e. in resonance with the V L_3 $2p_{3/2} \rightarrow 3d$ electronic transition). Second, we build a model of such an interface with the "simulation of electron spectra for surface analysis" software (SESSA),[2] using the same photon energy and collection geometry as available at the beamline 11.0.2 of the ALS (see below for further details). For the Mo:$BiVO_4$ modeling, the following parameters have been used: number of valence electrons/unit formula = 44; density of the photoemitting centers = $6.804 \cdot 10^{22}$ atoms·cm^{-3}; energy band gap = 2.45 eV. The thickness of a conformal carbon overlayer atop the Mo:$BiVO_4$ surface was thus varied until the C 1s/Bi 4f ratio matched the experimental value determined above (C 1s/Bi 4f = 0.02). This match was reached for a carbon layer equivalent thickness of 0.20 Å. Taking the *d*-spacing of 1 ML of $BiVO_4$ as half unit cell along the *b* direction (d = 5.76 Å)[1], the θ_C results therefore equal to 0.03 ML. Furthermore, the normalized C 1s core level peaks reported in **Figure S1b** taken during the AP-ResPES experiment show that the amount and chemical composition of the carbon surface contamination was stable throughout the investigation. The C 1s core level peak is characterized by two spectral contributions: the low binding energy (LBE) component, centered at 285.3 eV, can be attributed to adventitious carbon, whereas the high binding energy component (HBE, 287.2 eV) is associated to the presence of oxygenated carbon species.[3] The HBE/LBE intensity ratio was equal to about 1.14 throughout the AP-ResPES data collection.

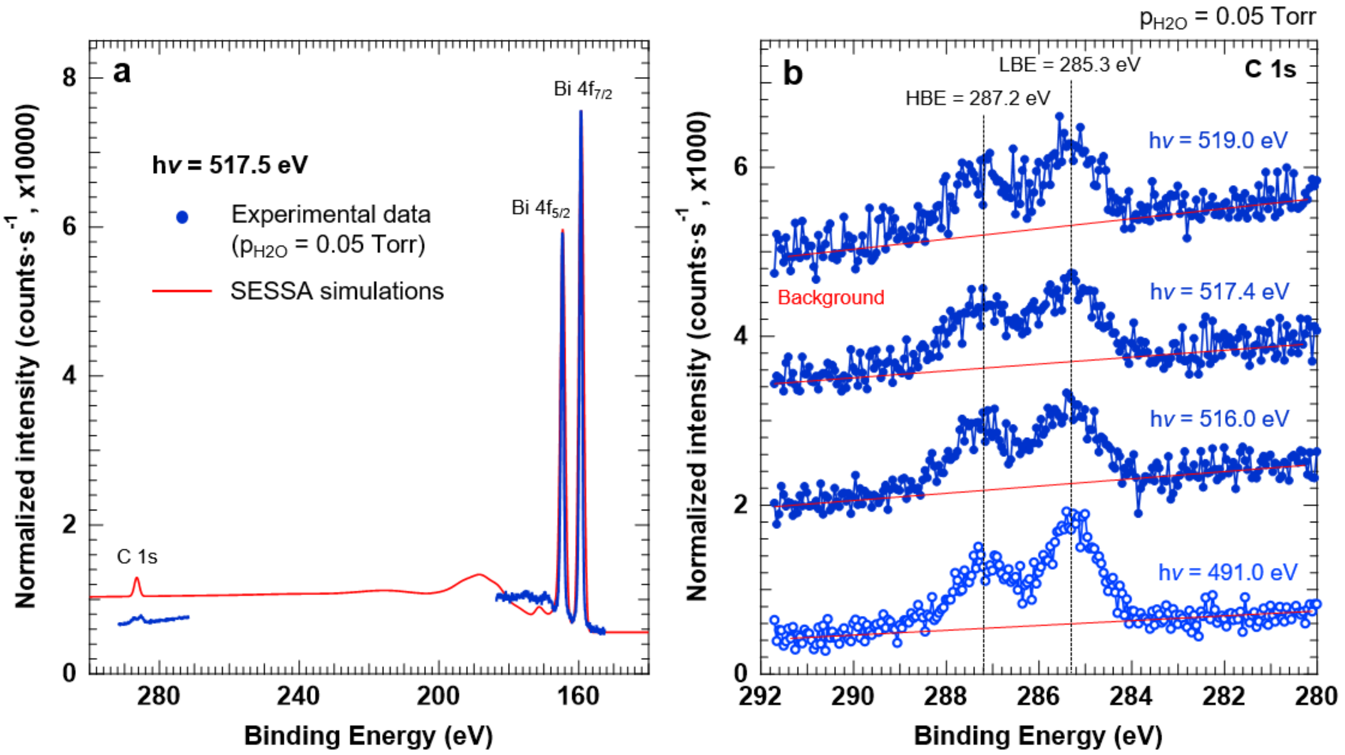

Figure S1. (a) Bi 4f and C 1s core level peaks (blue circles) taken at a water pressure of 0.05 Torr and at a photon energy (PE) of 517.4 eV (i.e. in resonance with the V L_3 $2p_{3/2}$ → 3d electronic transition). under dipolar approximation The outcome of the SESSA simulation is also reported (red line). The simulated spectrum was normalized to the experimental data by taking the intensity of the Bi $4f_{7/2}$ core level peak as reference; (b) C 1s core level peaks taken at different PEs and at a water pressure of 0.05 Torr. Note that the PEs of 516.0, 517.4, and 519.0 eV are in resonance with the V L_3 transition (LBE: low binding energy, HBE: high binding energy).

Soft X-ray photoelectron and resonant photoelectron spectroscopies

The end-station of beamline 11.0.2 at the Advanced Light Source (Lawrence Berkeley National Laboratory, Berkeley, U.S.A.) was used for AP-XPS and AP-ResPES measurements. The beamline is served by an elliptically-polarized undulator delivering photons from 90 eV up to ~1600 eV.[4,5] The end-station is equipped with a Near Ambient Pressure (NAP) PHOIBOS 150 electron analyzer (SPECS) placed at the magic angle (54.7°) between the X-ray polarization vector and the photoelectron momentum vector. For all measurements conducted at room temperature, the photoelectron take-off angle was set to 47° with respect to the surface normal, while the X-ray polarization was horizontal. The XPS data were acquired using a photoelectron kinetic energy (KE) of 200 eV, a step size of 0.05 eV and a pass energy of 20 eV. A 10 x 50 μm^2 (dispersive x non-dispersive) slit widths was used for all

measurements. Under these conditions, the total resolution (beamline and electron analyzer) was better than 100 meV at a photon energy of 735 eV at room temperature. The PEY-NEXAFS data (V $L_{2,3}$ and O K edges) were acquired using a photoelectron KE of 425 eV (λ_e ~ 13 Å[1]). The analyzer pass energy was set to 100 eV, providing an energy window of ±10 eV around the KE setpoint. The photon energy step was set to 0.1 eV, and the integration time was 1.0 s. The beamline resolution was better than 100 meV throughout both the V $L_{2,3}$ and O K energy ranges. The ResPES measurements across the V $L_{2,3}$ were conducted by acquiring VB spectra as the V L_3 edge was scanned. The photon energy was scanned in steps of 0.1 eV. The VB spectra were acquired with a photoelectron KE step of 0.05 eV and an integration time of 0.3 s.

Spectra Calibration

The binding energy (BE) scale for the core level and VB spectra was calibrated using the Au $4f_{7/2}$ photoelectron peak from a clean gold polycrystalline surface as a reference ($4f_{7/2}$ BE = 84.00 eV). This is valid as long as there is no charging. As we have shown in our previous work, we can exclude charging of the clean surface.[1] For the AP-ResPES data, the initial photon energy was calibrated by acquiring the Bi 4f spectrum of the Mo:$BiVO_4$ sample with the first and the second order light of the chosen photon energy; we assume the photon energy to be equal to the difference in KE of the Bi $4f_{7/2}$ peak for the spectra acquired with first and second order light. The final photon energy was calibrated by acquiring the Bi $4f_{7/2}$ spectrum with the 1st order of the final photon energy, and assuming that the KE difference between the initial and final Bi $4f_{7/2}$ spectra taken with 1st order light was equal to the difference in initial and final photon energy for the NEXAFS scan. BEs of the VB spectra were calibrated by acquiring different Bi $4f_{7/2}$ spectra at specific PEs, and using its calibrated BE as a reference value. The photon energies for the NEXAFS measurements were calibrated using the known XAS resonance positions of O_2 (g). For the latter, the corresponding NEXAFS spectrum was taken in PEY using a photoelectron KE of 425 eV and a pass energy of 100 eV. The O_2(g) pressure was set to 1 Torr.

Data Analysis

We performed quantitative XPS anlysis based on the background-subtracted integrated peak areas of each core level. Subtraction was done using Shirley backgrounds. Normalized areas were obtained by dividing the raw integrated core level peak area by the corresponding differential photoionization cross section and photon flux. The photon flux was measured using a Si photodiode (Opto Diode,

AXUV100G, 10 mm ×10 mm × 55 μm).

All core level fits reported in this work have been carried out after Shirley background subtraction, using a Doniach-Šunjić shape for the Au 4f and Bi 4f photoelectron peaks and symmetrical pseudo-Voigt functions (G/L ratio ranging from 85/15 to 75/25) for the V 2p, O 1s, and C 1s photoelectron peaks. During the fitting procedure, the Shirley background was optimized together with the spectral components in order to increase the precision and reliability of the fitting procedure.[6–8] The VB was fitted by subtracting a Tougaard background[6] and using symmetrical Voigt functions (G/L ratio ranging from 85/15 to 95/5).

Calculation Methods

Enumeration of configurations

At the (010) surface, the surface V and Bi atoms are respectively four-fold and six-fold coordinated by O atoms; in the bulk, the V atoms are also four-fold coordinated but the Bi atoms are eight-fold coordinated. In our 2× 2× 2 supercell, water molecules may be adsorbed at four possible Bi atoms and four possible V atoms at the surface. Water molecules were initialized to be adsorbed to a surface Bi atom via their O atom, thus increasing the coordination number of the Bi atom to seven (see **Figure 1** of main text). Water molecules are expected to be more favorably adsorbed on Bi atoms when the oxygen points at Bi, as surface Bi are under-coordinated relative to the bulk with six surrounding oxygen atoms; in contrast, the surface V atoms are four-fold coordinated at the surface and in the bulk. This expectation was corroborated by our own calculations and is consistent with previous computational studies.[9,10]

To mimic electron-doped surface configurations, we added excess electronic charge to the supercell, which experimentally may originate from Mo-doping found in our samples or oxygen vacancies that intrinsically occur in the material. Specifically, in our 192-atom symmetric slabs, we introduced one electron per surface for a total of two excess electrons. It is well known that small polarons form in $BiVO_4$.[11–14] In order to encourage the formation of polarons (which localize on V sites to nominally reduce the V^{5+} to V^{4+}), we extended the V-O bonds of a surface VO_4 tetrahedron by 3% and initialized the same surface V site with a small but non-zero magnetic moment (denoted as a pink atom in **Figure 1** of the main text).

We introduced water species to either the bare surface (configurations denoted as s1) or the surface with electron doping to form a V^{4+} polaron (denoted as s2). From our measurements of sub-monolayer coverage with partial pressure of water, we observed that hydroxylation occurs via island growth on the (010) surface. The adsorbed water in these islands may take on several orientations. We expect the water to orient in such a way that its hydrogen atoms are near the oxygen on a VO_4 tetrahedron. Thus, for any single surface VO_4 tetrahedron, there may be one or two water molecules oriented towards it. In order to computationally investigate these configurations, we introduced two water molecules to each exposed surface of the slab. As shown in **Figure 1** of the main text, each central (pink) VO_4 tetrahedron is either single- or double-hydroxylated by water. That is, a surface VO_4 tetrahedron is considered single-

hydroxylated if only one O atom in the tetrahedron is (hydrogen) bonded with the H atom of water adsorbed via the O atom on neighboring surface Bi atoms. Similarly, a surface VO_4 tetrahedron is double-hydroxylated if two of the O atoms in the tetrahedron are (hydrogen) bonded with the H atoms of adsorbed water.

We further differentiated whether the adsorbed water species was in the molecular form or dissociated form. The bonds involved in the hydroxylation process could be either a hydrogen bond or an O-H bond in the molecular and dissociated cases, respectively. For configurations with dissociated water, we initialized the surface such that the water was split as neutral OH and H species; the OH species are adsorbed on the surface Bi sites and the H species are adsorbed on surface O to form additional OH species.

Naming convention

In our naming convention, we specify whether the configuration involves adsorption of water molecules oriented towards different (s3) or the same (s4) surface VO_4, and denote the type of water ms oriented towards the same surface VO_4 (indicated by "s4"). We further specify what kind of doping, if any, was used. For example, the s2e configuration had electron doping of one e- per surface, while the s2Mo configuration had explicit Mo doped in the bulk-like region of the slab. Here, "s2" indicates a configuration where the V-O bonds of a surface VO_4 tetrahedron are extended to induce the formation of an electron polaron, as described above. All s3 and s4 configurations have electron doping, unless otherwise indicated by a tilde for no excess electrons (as in $\widetilde{\mathrm{s3}}$mm) or an "Mo" suffix for explicit Mo doping (as in s4mm-Mo). Once an initial configuration was established, we allowed the internal coordinates of the slab to relax. We found that some configurations were difficult to fully relax, predominantly because of water-related species drifting and/or rotating above the surface. We were able to relax the s4dm, s4dmm, s4ddm, and $\widetilde{\mathrm{s4}}$dd configurations bringing atomic forces below 700 meV/Å; the $\widetilde{\mathrm{s3}}$mm, $\widetilde{\mathrm{s4}}$mm and s3dm configurations were relaxed to forces within around 300 meV/Å with total energies converged to within 5 meV. All other configurations were relaxed until forces were about 100 meV/Å.olecules and the number of water molecules present at each exposed surface. For example, the "s4dmm" configuration denotes one dissociated water (indicated by "d") and two molecular water molecules (indicated by "mm") initially adsorbed, and with one dissociated and one molecular water species.

Table S1: Notation and description of configurations tested in our study, noting whether the adsorbed water species are in molecular or dissociated forms. All configurations starting with s1 and s2 are the pristine $BiVO_4$ surface, with s2e referring to those with a polaron localized on a surface VO_4 tetrahedra and s2 referring to the configuration with the s2e structure but without the surface polaron. All configurations starting with s3 and s4 have adsorbed water and a surface polaron localized on a VO_4 tetrahedra; s3 = one water oriented towards a particular VO_4, s4 = two waters oriented towards a particular VO_4. All s3 and s4 configurations all have one excess electron per surface, unless otherwise noted by an "-Mo" suffix or tilde. The tilde indicates no electron doping whereas the "Mo" suffix indicates a Mo_V substitutional defect was included. Adsorbed molecular water forms hydrogen bonds, while adsorbed dissociated water forms hydroxyl groups.

	**Polaron type**	**Doping model**	**# molec**	**# dissoc**	**# of waters oriented towards a particular VO_4**
**s1**	—	—	0	0	—
**s1e**	—	excess e-	0	0	—
**s2**	surface	—	0	0	—
**s2e**	surface	excess e-	0	0	—
**s2e-bulk**	bulk	excess e-	0	0	—
**s2Mo**	surface	Mo	0	0	—
$\widetilde{\text{s3}}$**mm**	surface	—	2	0	1
**s3mm-Mo**	surface	Mo	2	0	1
$\widetilde{\text{s3}}$**dd**	surface	—	0	2	1
**s3dd**	surface	excess e-	0	2	1
**s3dd-Mo**	surface	Mo	0	2	1
$\widetilde{\text{s4}}$**mm**	surface	—	2	0	2
**s4mm**	surface	excess e-	2	0	2
**s4mm-Mo**	surface	Mo	2	0	2
$\widetilde{\text{s4}}$**dd**	surface	—	0	2	2
**s4dd**	surface	excess e-	0	2	2
**s4dd-Mo**	surface	Mo	0	2	2
**s3dm**	surface	excess e-	1	1	1
**s4dm**	surface	excess e-	1	1	2
**s3dmm**	surface	excess e-	2	1	1
**s4dmm**	surface	excess e-	2	1	2
**s3ddm**	surface	excess e-	1	2	1
**s4ddm**	surface	excess e-	1	2	2

Additional results of calculated configurations

We first considered the adsorption of water molecules in the absence of electron doping. The corresponding configurations are the single-hydroxylated $\widetilde{s3}$mm and double-hydroxylated $\widetilde{s4}$mm. In general, the adsorption of molecular water on the Bi site in the absence of excess charge was found to be favorable, leading to an average stabilization of 0.54 (0.45) eV/water molecule for $\widetilde{s3}$mm ($\widetilde{s4}$mm). This stabilization energy was comparable to the range reported in the literature (0.48 and 0.58 eV[9,10]). In neither the $\widetilde{s3}$mm or the $\widetilde{s4}$mm configuration did we find an interband state in the band gap. The distances between the surface Bi and adsorbed water molecules were 2.64 Å for $\widetilde{s3}$mm and 2.62 Å for $\widetilde{s4}$mm, consistent with previous literature values based on first-principles molecular dynamics simulations (2.65-2.97 Å[9]). A summary of the bond lengths and angles at the surface for selected configurations is reported in **Table S2**.

Since the adsorption of molecular water either did not result in a defect state or led to a configuration which was not stable, we turned to configurations involving dissociated water moieties in the single-hydroxylated (s3dd) and double-hydroxylated (s4dd) forms. We note again that in these configurations electron doping was added to our supercell. We found that the s3dd configuration was 140 meV more stable than the s4dd configuration, and 435 meV more stable compared to the bare surface with a surface polaron plus the equivalent number of isolated water molecules. This suggests that it is more favorable to adsorb dissociated water molecules in the presence of a surface polaron. The relative stability of the s3dd and s4dd configurations relative to a bare surface with a surface polaron is consistent with the analysis of our ResPES results, which suggest that hydroxylation of the surface leads to additional localization of charge (small electron polarons) at the surface. The distances between the surface Bi and hydroxyls from dissociated water were 2.17 Å for s3dd and 2.18 Å for s4dd; these distances are up to 0.4 Å smaller than that for any of the configurations involving molecular water adsorption on the bare surface.

We subsequently considered configurations involving combinations of dissociated and molecular adsorbed water. These included configurations with a dissociated moiety plus an adsorbed water molecule (dm); one dissociated plus two molecular units (dmm), and two dissociated and one molecular unit (ddm). In these configurations, the surface V^{4+} polaron was always hydroxylated by at least one dissociated water. The V^{4+} polaron was then hydroxylated by a second water in either molecular or

dissociated form to create the s4 configurations. In configurations with three water species, the third molecular water was initialized on a third Bi neighbor of the VO_4 tetrahedron, and it was not hydrogen bonded to the VO_4 tetrahedron (see **Figure S2** in the SI for a full list of configurations tested).

In these instances of mixed molecular and dissociated adsorption on nearest-neighbor or next-nearest neighbor sites, all water molecules that were initially adsorbed on Bi subsequently desorbed from Bi upon relaxation. For all dissociated moieties, the OH and H species remained adsorbed to the surface Bi and O atoms, respectively. We note that the initial state we chose for the water molecules impacted the configuration found upon relaxation. If the molecular water was initialized to be hydrogen bonded to the V polaron, it remained hydrogen bonded despite having desorbed from Bi. Meanwhile, if the molecular water was initialized without a hydrogen bond to the V polaron, it desorbed from the surface completely. For example, in the s4dmm configuration with two water molecules, both of them desorbed from the surface Bi atoms. However, one molecular water remained hydrogen bonded to the VO_4 tetrahedron while the other desorbed completely.

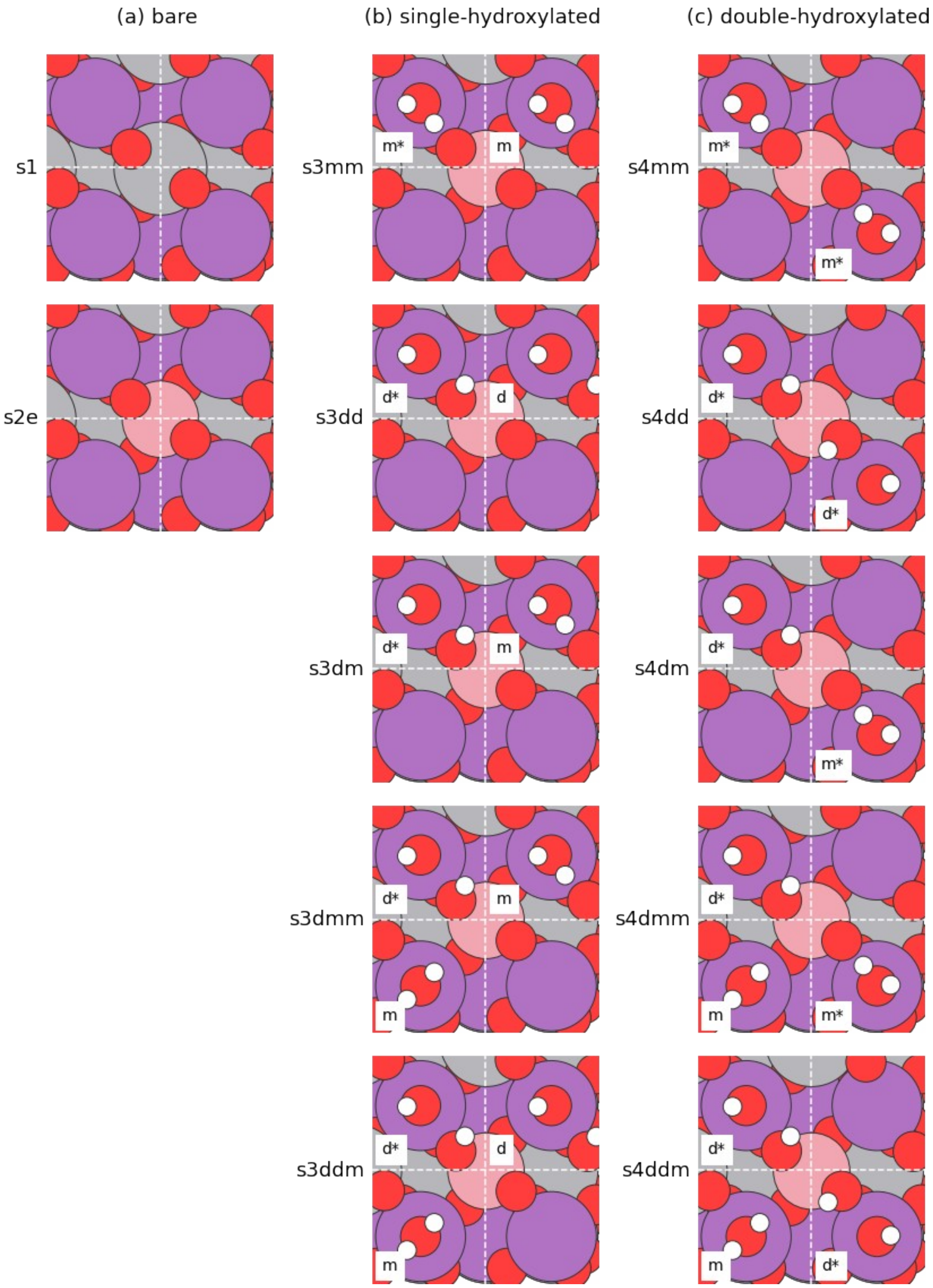

Figure S2: Summary of structural variations considered in our calculations. Plots shown are the initial configurations of (a) the bare surface, (b) the single-hydroxylated surfaces and (c) the double-hydroxylated surfaces. Neighboring Bi atoms (purple) of the central VO_4 in contact with an adsorbed water molecule are labeled either 'd' for dissociated or 'm' for molecular. An asterix (*) indicates that the adsorbed water was initialized to be hydrogen bonded to an O atom in the central VO_4 tetrahedron. V atoms initialized to favor localization of polarons are pink, remaining V atoms are gray, oxygen atoms are red and hydrogen atoms are white.

In general, the presence of adsorbed species may alter the surface electronic structure (**Table S2**). Notably, we see variations of approximately 0.14 eV in the band gap for the configurations considered here. Recalling that there are four possible adsorption Bi sites at the surface, all configurations involving adsorbed water involve at minimum 50% surface coverage. For the s3dd configuration with 50% surface coverage, the calculated Fermi level is 0.1 eV closer to the CBM compared to that of the undoped unhydroxylated $BiVO_4$ (010) surface, which gives an approximate error bar in our identification of the relative position of the defect state. Single-particle energy levels for determining the relative positions of defect polaron states were sampled at the Γ point; differences on the order of at most 5 meV in the eigenvalues of the CBM, VBM, and defect state occurred when sampling the Brillouin zone using a denser 10 × 10 × 1 *k*-point grid. As our main comparison is the relative position of the defect state, we expect the general findings of our calculations carried out with only the Γ point to hold in comparison to using denser *k*-point grids.

TABLE S2. Summary of the relative position of defect states with respect to the VBM for experiment (exp.) and selected computed configurations. The configurations where the polaron forms on a surface V atom are indicated as "surf"; similarly the configurations in which the polaron forms in the bulk-like region of the slab, are indicated as "in bulk" (see main text and **Figure S2** for full description of configurations). Band gaps are computed using only the Γ point to sample the supercell Brillouin zone.

	**Bare**			**Hydroxylated**					
	exp	s2e	s2mo	exp	$\widetilde{\mathrm{s3}}$mm	$\widetilde{\mathrm{s4}}$mm	s3dd	s4dd	s3dd-mo
Polaron type	-	surf	In bulk		surf	surf	surf	surf	In bulk
# waters oriented toward a given VO_4					1	2	1	2	1
Molecular or dissociated					molec	molec	dissoc	dissoc	dissoc
Band gap (eV)	2.60	2.34	2.28	2.60	2.36	2.35	2.33	2.20	2.15
Defect level (eV from the VBM)	0.90	1.72	1.26	0.50	-	-	1.09	0.04	1.07

While our calculations qualitatively corroborate our measurements, there is a quantitative difference, particularly regarding the relative position of the polaron level. Our calculated polaron levels are farther from the VBM compared to those found in our AP-ResPES measurements. We consider two possible contributing factors: the level of theory chosen and (mainly) finite temperature corrections not captured in our DFT calculations. We tested the robustness of DFT+U by performing a one-shot (i.e., single self-consistent cycle) calculation at a higher level of theory using the dielectric-dependent hybrid (DDH) functional[15] on the s3dd configuration. In DDH, the mixing fraction of Hartree-Fock exact

exchange was chosen as the inverse of the high-frequency dielectric constant. The mixing fraction used for $BiVO_4$ was $\alpha = 1/\varepsilon_\infty = 1/6.9 = 0.145$.[16,17] Interestingly, we found that the defect polaron state using DDH was proportionally as far from the VBM as it was using DFT+U; that is, the defect state for both DDH and DFT+U levels of theory was about 45% of the band gap above the VBM (see **Figure 5** of the main text).

We seek to estimate to temperature re-normalization effects on the band edges to understand the discrepancy between theory and experiments in the relative position of the defect state. We estimate the influence of finite temperature on the relative positions of the band edges and defect state by drawing upon previous molecular dynamics calculations of $BiVO_4$ from Wiktor and Pasquarello.[13,14] As shown in our previous study of the $BiVO_4$ (010) surface,[11] including corrections based on path-integral molecular dynamics simulations of the bulk[14] to our band alignments based on the DDH functional leads to the most robust agreement with experiment compared to PBE or PBE+U; finite-temperature renormalization and nuclear quantum effects contribute the most to this correction, followed by spin-orbit coupling and excitonic effects. We estimated[11] a renormalization of conduction band edge to fall by about 0.54 eV and the valence band edge to rise by about 0.61 eV when including finite temperature, nuclear quantum effects, excitonic effects, and spin-orbit coupling. Wiktor and Pasquarello[13] furthermore found that on an absolute scale the electron polaron level shifts down (closer to the VBM) by around 0.16 eV when considering finite temperature effects for the $BiVO_4$ (010) surface. Applying these corrections independently to the band edges and defect level to the s3dd configuration, respectively, using DDH (see **Figure 5d** of the main text), we estimate the relative position of the defect state in the s3dd configuration to be approximately 0.6 eV above the VBM, which is in reasonable agreement with the relative position of the polaron state in our AP-ResPES measurements.

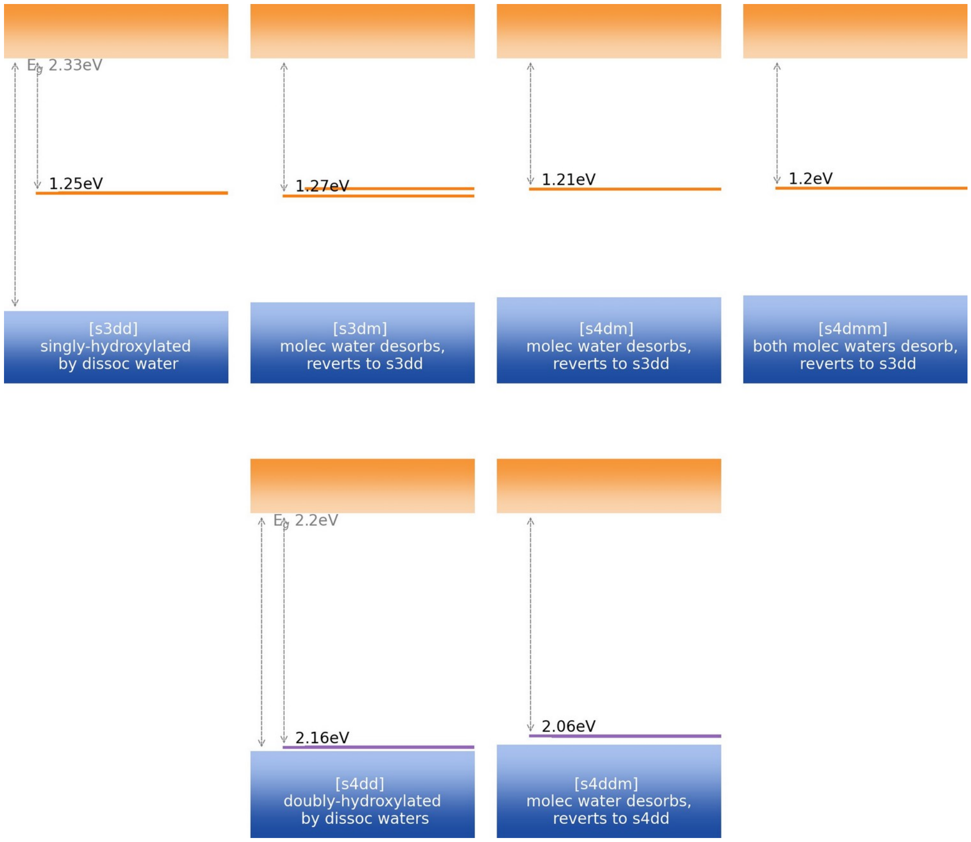

Figure S3: Band diagrams showing relative positions of defect states after desorption of molecular water for the mixed dissociated-molecular configurations in comparison to configurations with only dissociated water moieties adsorbed to the surface. Configurations showing two very close, but separate defect levels are due to the slight asymmetry between the two surfaces of the slab after desorption. The positions of the single-particle energies are referenced to the conduction band minimum. Computed band gaps for the various configurations are 2.33eV (s3dd), 2.26eV (s3dm), 2.21eV (s4dm), 2.19eV (s4dmm), 2.20eV (s4dd), 2.14eV (s4ddm).

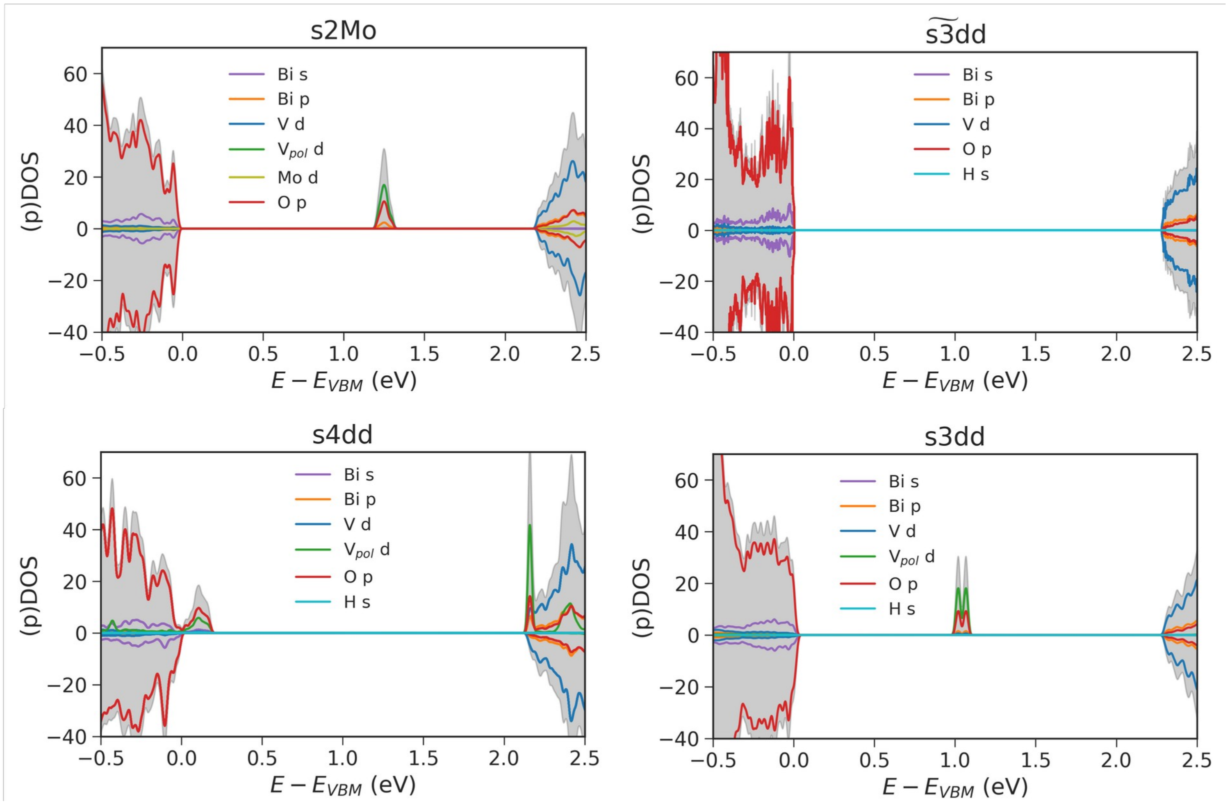

Figure S4: Projected density of states for selected configurations computed using DFT+U. Where relevant, the contribution from the surface V site where the electron polaron localizes is indicated as V_{pol}.

Table S3: Representative distances and angles for relaxed configurations. For the V-O_{slab} distances, the top bond lengths correspond to bonds of the topmost oxygen and the bottom bond lengths correspond to bonds of the next layer of oxygen VO_4 site shown in **Figure 1**. Adsorbed water molecules were initialized to be 2.26 Å away from Bi. Subscripts *d* and *m* indicate dissociated or molecular water species, respectively. Subscripts "slab" and "ad" indicate whether the atomic site was located in the slab of $BiVO_4$ or related to an adsorbed species. Note (*): for the s3mm, s4mm, s3dmm and s3ddm configurations, we could not reach electronic self-consistency.

	**Distances (Å)**			**Bond angles (°)**		
	V-O_{slab}†	Bi-O_{ad}	O_{slab}-H_{ad}	V-O_{slab}-Bi	V-O_{slab}-H_{ad}	H_2O (ad)
$\widetilde{s3}$mm	1.67, 1.69<br>1.82, 1.82	2.66	1.94	120.52	161.53	108.31
$\widetilde{s3}$dd	1.81, 1.63<br>1.76, 1.75	2.16	0.98	124.10	133.93	159.5
$\widetilde{s4}$mm	1.67, 1.67<br>1.81, 1.81	2.62	1.95	118.49	159.30	108.67
s3mm*	1.72, 1.70<br>1.85, 1.85	2.66	1.94	120.52	161.53	108.31
s3dd	2.05, 1.64<br>1.89, 1.94	2.17	0.97	124.77	114.51	171.63
s4mm*	1.71, 1.71<br>1.84, 1.84	2.62	1.95	118.49	159.3	108.67
s4dd	1.90, 1.90<br>1.73, 1.73	2.18	0.97	119.85	127.47	172.93
s3dm	2.03, 1.64<br>1.89, 1.92	2.17_d 2.76_m	0.97_d 3.70_m	126.33	116.59_d	102.77_m
s4dm	2.03, 1.65<br>1.89, 1.91	2.17_d 2.88_m	0.97_d 1.78_m	117.59	126.79_d	105.46_m
s4dmm	1.98, 1.68<br>1.89, 1.87	2.17_d 3.03_m 3.21_m	0.97_d 1.79_m 4.28_m	122.36	124.54_d	105.63_m
s4ddm	1.89, 1.90<br>1.78, 1.78	2.19_d 2.19_d 3.12_m	0.98_d 0.98_d 4.24_m	124.40	132.52_d	102.83_m
s2e	1.75, 1.75<br>1.90, 1.90	-	-	123.80	-	-
s1	1.67, 1.67<br>1.82, 1.82	-	-	123.28	-	-

† V-O bonds in the bulk are 1.73 and 1.75 Å

References

(1) Favaro, M.; Uecker, R.; Nappini, S.; Píš, I.; Magnano, E.; Bluhm, H.; van de Krol, R.; Starr, D. E. Chemical, Structural, and Electronic Characterization of the (010) Surface of Single Crystalline Bismuth Vanadate. *J. Phys. Chem. C* **2019**, *123* (13), 8347–8359. https://doi.org/10.1021/acs.jpcc.8b09016.

(2) Smekal, W.; Werner, W. S. M.; Powell, C. J. Simulation of Electron Spectra for Surface Analysis (SESSA): A Novel Software Tool for Quantitative Auger-Electron Spectroscopy and X-Ray Photoelectron Spectroscopy. *Surf. Interface Anal.* **2005**, *37* (11), 1059–1067. https://doi.org/10.1002/sia.2097.

(3) Nemšák, S.; Shavorskiy, A.; Karslioglu, O.; Zegkinoglou, I.; Rattanachata, A.; Conlon, C. S.; Keqi, A.; Greene, P. K.; Burks, E. C.; Salmassi, F.; Gullikson, E. M.; Yang, S.-H.; Liu, K.; Bluhm, H.; Fadley, C. S. Concentration and Chemical-State Profiles at Heterogeneous Interfaces with Sub-Nm Accuracy from Standing-Wave Ambient-Pressure Photoemission. *Nat. Commun.* **2014**, *5* (1), 5441. https://doi.org/10.1038/ncomms6441.

(4) Ogletree, D. F.; Bluhm, H.; Lebedev, G.; Fadley, C. S.; Hussain, Z.; Salmeron, M. A Differentially Pumped Electrostatic Lens System for Photoemission Studies in the Millibar Range. *Rev. Sci. Instrum.* **2002**, *73* (11), 3872–3877. https://doi.org/10.1063/1.1512336.

(5) Bluhm, H. Photoelectron Spectroscopy of Surfaces under Humid Conditions. *J. Electron Spectrosc. Relat. Phenom.* **2010**, *177* (2–3), 71–84. https://doi.org/10.1016/j.elspec.2009.08.006.

(6) Tougaard, S. Practical Algorithm for Background Subtraction. *Surf. Sci.* **1989**, *216* (3), 343–360. https://doi.org/10.1016/0039-6028(89)90380-4.

(7) Evans, S. Curve Synthesis and Optimization Procedures for X-Ray Photoelectron Spectroscopy. *Surf. Interface Anal.* **1991**, *17* (2), 85–93. https://doi.org/10.1002/sia.740170204.

(8) Muñoz-Flores, J.; Herrera-Gomez, A. Resolving Overlapping Peaks in ARXPS Data: The Effect of Noise and Fitting Method. *J. Electron Spectrosc. Relat. Phenom.* **2012**, *184* (11–12), 533–541. https://doi.org/10.1016/j.elspec.2011.08.004.

(9) Oshikiri, M.; Boero, M. Water Molecule Adsorption Properties on the $BiVO_4$ (100) Surface. *J. Phys. Chem. B* **2006**, *110* (18), 9188–9194. https://doi.org/10.1021/jp0555100.

(10) Crespo-Otero, R.; Walsh, A. Variation in Surface Ionization Potentials of Pristine and Hydrated $BiVO_4$. *J. Phys. Chem. Lett.* **2015**, *6* (12), 2379–2383. https://doi.org/10.1021/acs.jpclett.5b00966.

(11) Wang, W.; Strohbeen, P. J.; Lee, D.; Zhou, C.; Kawasaki, J. K.; Choi, K.-S.; Liu, M.; Galli, G. The Role of Surface Oxygen Vacancies in $BiVO_4$. *Chem. Mater.* **2020**, *32* (7), 2899–2909. https://doi.org/10.1021/acs.chemmater.9b05047.

(12) Wiktor, J.; Pasquarello, A. Electron and Hole Polarons at the $BiVO_4$ –Water Interface. *ACS Appl. Mater. Interfaces* **2019**, *11* (20), 18423–18426. https://doi.org/10.1021/acsami.9b03566.

(13) Wiktor, J.; Ambrosio, F.; Pasquarello, A. Role of Polarons in Water Splitting: The Case of $BiVO_4$. *ACS*

Energy Lett. **2018**, *3* (7), 1693–1697. https://doi.org/10.1021/acsenergylett.8b00938.

(14) Wiktor, J.; Reshetnyak, I.; Ambrosio, F.; Pasquarello, A. Comprehensive Modeling of the Band Gap and Absorption Spectrum of $BiVO_4$. *Phys. Rev. Mater.* **2017**, *1* (2), 022401(R). https://doi.org/10.1103/PhysRevMaterials.1.022401.

(15) Skone, J. H.; Govoni, M.; Galli, G. Self-Consistent Hybrid Functional for Condensed Systems. *Phys. Rev. B* **2014**, *89* (19), 195112. https://doi.org/10.1103/PhysRevB.89.195112.

(16) Kim, T. W.; Ping, Y.; Galli, G. A.; Choi, K.-S. Simultaneous Enhancements in Photon Absorption and Charge Transport of Bismuth Vanadate Photoanodes for Solar Water Splitting. *Nat. Commun.* **2015**, *6*, 8769. https://doi.org/10.1038/ncomms9769.

(17) Seo, H.; Ping, Y.; Galli, G. Role of Point Defects in Enhancing Conductivity of $BiVO_4$. *Chem. Mater.* **2018**, *30* (21), 7793–7802. https://doi.org/10.1021/acs.chemmater.8b03201.
